# Structurally informed resting-state effective connectivity recapitulates cortical hierarchy

**DOI:** 10.1101/2024.04.03.587831

**Published:** 2024-04-04

**Authors:** Matthew D. Greaves, Leonardo Novelli, Adeel Razi

**Affiliations:** 1 Turner Institute for Brain and Mental Health, School of Psychological Sciences, Monash University, Clayton, 3800, Australia; 2 Monash Biomedical Imaging, Monash University, Clayton, 3800, Australia; 3 Wellcome Centre for Human Neuroimaging, University College London, London, WC1N 3AR, United Kingdom; 4 CIFAR Azrieli Global Scholars Program, CIFAR, Toronto, M5G 1M1, Canada

## Abstract

Interregional brain communication is mediated by the brain’s physical wiring (i.e., structural connectivity). Yet, it remains unclear whether models describing directed, functional interactions between latent neuronal populations—effective connectivity—benefit from incorporating macroscale structural connectivity. Here, we assess a hierarchical empirical Bayes method: structural connectivity-based priors constrain the inversion of group-level resting-state effective connectivity, using subject-level posteriors as input; subsequently, group-level posteriors serve as empirical priors for re-evaluating subject-level effective connectivity. This approach permits knowledge of the brain’s structure to inform inference of (multilevel) effective connectivity. In 17 resting-state brain networks, we find that a positive, monotonic relationship between structural connectivity and the prior probability of group-level effective connectivity generalizes across sessions and samples. Providing further validation, we show that inter-network differences in the coupling between structural and effective connectivity recapitulate a well-known unimodal-transmodal hierarchy. Thus, our results provide support for the use of our method over structurally uninformed alternatives.

## Introduction

How the structural connectivity of the human brain gives rise to its functional dynamics, is an enduring question in neuroscience. *In vivo*, macroscale structural connectivity is often inferred via tractography applied to diffusion-weighted imaging (DWI): a magnetic resonance imaging (MRI) acquisition that estimates the directional diffusivity of water molecules.^1,2^ The brain’s functional dynamics, on the other hand, are often summarized in terms of (undirected) functional connectivity: statistical dependencies between time-series data encoding, for example, changes in blood-oxygen-level-dependent (BOLD) signal intensity detected via functional MRI (fMRI).^3,4^ Although much research has focused on the issue of how to understand the relationship between structural and functional connectivity,^5,6^ far less attention has been paid to the issue of how structural connectivity constrains the directed interactions—directed connectivity—that gives rise to functional connectivity.

In the context of fMRI, directed connectivity models tend to be partitioned into models of effective and *directed* functional connectivity, depending on whether they attempt to disentangle hemodynamic effects from the activity of latent neuronal populations assumed to underlie the generation of BOLD time-series data.^4,7,8^ For example, dynamic causal modelling (DCM)^†^ is used to infer effective connectivity—the directed influence that one neuronal population exerts on another—from BOLD time-series data via the inversion of a biophysical generative model that incorporates a hemodynamic function.^9,10^ In contrast, multivariate autoregressive (MAR) models, which can, like DCM, be formulated to generate data via a state-space approach, are primarily utilized to infer directed functional connectivity: a map of predictive relationships describing how well one BOLD time series can usefully forecast another.^11,12^

Broadly, prior research has taken three approaches to examining how structural connectivity constrains directed connectivity. First, a Bayesian approach that involves constraining the inversion of generative models with structural connectivity-based (i.e., structure-based) priors.^13–22^ Second, a mechanistic approach, via which structural connectivity is incorporated directly into a generative model’s equations (rather than being incorporated into priors over the equations’ parameters).^23–25^ Finally, a data-driven machine learning (ML) approach, which leverages various ML techniques to infer a map of directed interactions from both structural and functional connectivity taken together.^26–29^

The first (Bayesian) approach has received the most attention, with previous research demonstrating that, compared to models with generic, hand-coded priors, models that incorporate structure-based priors yield higher model evidence.^13,18,19^ These improvements notwithstanding, there is a dearth of studies attempting to assess the reliability or validity of this approach and, to our knowledge, only a single study has examined structurally informed models of resting-state effective connectivity in humans.^21^ The lack of any out-of-sample validation, in particular, represents a significant limitation, as empirical Bayes-like methods—those that leverage within-sample data to constrain the estimation of model parameters—can be vulnerable to overfitting.^30,31^ Thus, the question which begs investigation is whether the assumed functional form of the relationship between structural and effective connectivity—i.e., the strength of the former scales the probability of the latter^17^—holds across resting-state networks, timepoints and samples.

**Fig 1.**
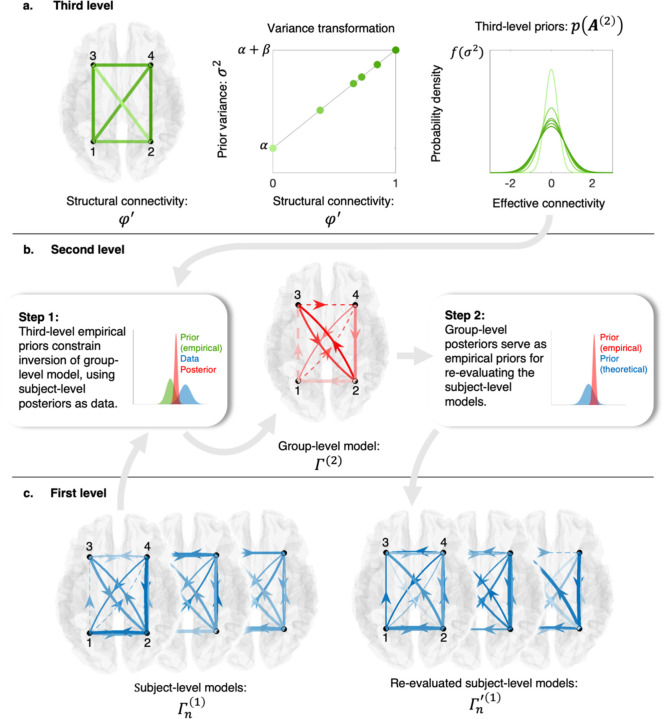
Schematic representation of the two-step, hierarchical empirical Bayes model used in this study. The three rows represent the levels of the hierarchical model, with mathematical expressions consistent with those used in equations 3–4. Inset text boxes and arrows describe the processes via which empirical data are integrated at multiple levels of the hierarchy. The left inset box displays a plot containing three probability density functions: a structure-based prior (in green), data (in blue), and the resulting posterior (in red), illustrating the Bayesian updating of priors. The right inset box shows a similar plot, in which the red probability density function represents the updated (empirical) prior used for re-evaluating subject-level models. This prior is contrasted with the initial (theoretical) prior (in blue), with the difference in precision reflecting a search for more precise or reduced models. **a** At the third level, structure-based priors are furnished by linearly mapping (normalized) structural connectivity weights to variances of zero-mean Gaussian priors. In this row, the undirected structural connectivity graph is formatted such that the weights are represented by the saturation of the color, with more saturated lines indicating stronger connectivity. **b** At the second level, a group-level model is furnished. Here, we represent a (directed) effective connectivity graph that is formatted such that solid (dashed) lines represent positive (negative) connections, with line weight representing the connection strength—the higher the weight the stronger the connection—and saturation of the color representing the precision of the estimate (with more saturated lines indicating more precise estimates). **c** At the first level, subject-level models—i.e., dynamic causal models—both serve as data (step 1), and are re-evaluated (step 2). The subject-level effective connectivity graphs are formatted per the conventions employed for the group-level effective connectivity graph.

Addressing the identified issues, this study assesses the reliability and validity of a two-step, hierarchical empirical Bayes procedure wherein structure-based priors inform the inversion of group-level (resting-state) effective connectivity estimates, which serve as empirical priors for re-evaluating subject-level effective connectivity (Fig. 1). We find a consistent, monotonic relationship between structural and effective connectivity across 17 different networks, as defined in Shaefer and colleagues’ cortical atlas.^32^ In tests of reliability and validity, we establish that network-specific relationships between structural and effective connectivity generalize out of session and out of sample. Finally, we explore inter-network differences in the coupling between structural and effective connectivity and show that these differences align with an established cortical hierarchy.^33–37^ Based on this reliability and validity, we recommend this hierarchical empirical Bayes procedure over structurally uniformed approaches to inferring effective connectivity.

## Results

### Face validity

In an exploratory, face-validation phase, for each network, we inverted a null (structurally uninformed) group-level effective connectivity model—the second-level model in the hierarchical empirical Bayes procedure (see Fig. 1b)—using generic, hand-coded priors, and DCM posteriors as input. Then, we explored different parameter regimes for the intercept and slope of a (structural connectivity to) variance transformation function (see Fig. 1a), using Bayesian model reduction (BMR) to score resultant (structurally informed) group-level effective connectivity models’ evidence, relative to the evidence for the null model. This enabled the derivation of a Bayesian model-average (BMA)^‡^ variance transformation per brain network. We refer the reader to the Methods section for detailed procedures.

**Fig 2.**
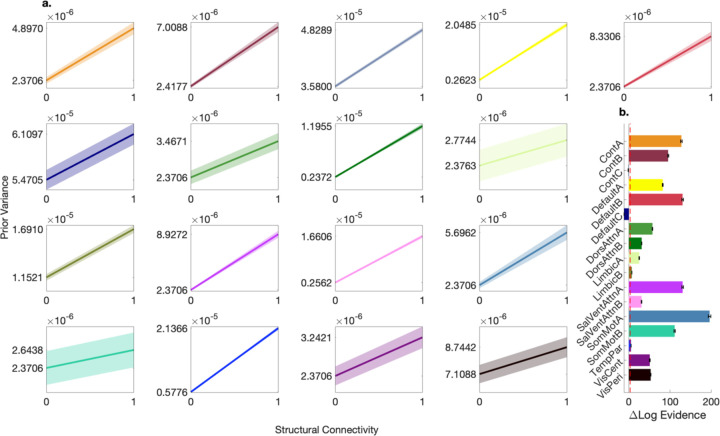
Bayesian model-average variance transformation for the 17 Schaefer networks (100 subjects). **a** Subplots show Bayesian model-average variance transformations with 95% confidence envelopes for each structurally informed group-level effective connectivity model—at the second level of the hierarchical empirical Bayes procedure—evaluated in the exploratory, face-validation phase. The x-axis represents normalized structural connectivity, while the y-axis represents the prior variance. Tick marks on the y-axis indicate the value of the intercept and the sum of the intercept and slope parameter. We refer the reader to the next panel for color legend. **b** This panel shows additional results for the exploratory, face-validation phase. The bar plot shows the mean log evidence for each structurally informed group-level effective connectivity model relative to null (structurally uninformed) group-level effective connectivity models with generic, hand-coded priors. Error bars show the 95% confidence interval. Note a dashed red line indicates a relative log evidence (or log Bayes factor) of 3, considered strong evidence against the null.38 Tick marks on the y-axis show Schafer network abbreviations (we refer the reader to the network parcellation subsection of the Methods section for network nomenclature). The color scheme for the bars follows a standardized palette and is applied to the plots in the first panel.

Fig. 2a illustrates that, despite the assessment of parameter regimes resulting in both flat and negative variance transformations, the BMA variance transformation for each brain network was a positive, monotonic function. In other words, positive variance transformations yielded higher (relative) model evidence. Note that the scaling of these BMA variance transformations was on the order of 10^−5^ and 10^−6^, indicating that prior variances were sensitive to subtle changes in structural connectivity; a sensitivity that appeared to be relatively precise since the width of the 95% confidence envelopes were stable across the domain of the functions (the [0,1] horizontal range representing the normalized structural connectivity). Fig. 2b shows that for most networks, on average, the introduction of structure-based priors in the exploratory, face-validation phase substantially increased model evidence, with a relative log evidence (or log Bayes factor) exceeding 3, providing strong support for these alternative models.^38,39^ Regarding one control (ContC) and one default mode (DefaultC) network, however, there was negative evidence for alternative models: for these networks, on average, relative log evidence decreased with the addition of structure-based priors.

### Within-subjects test-retest reliability

**Fig 3.**
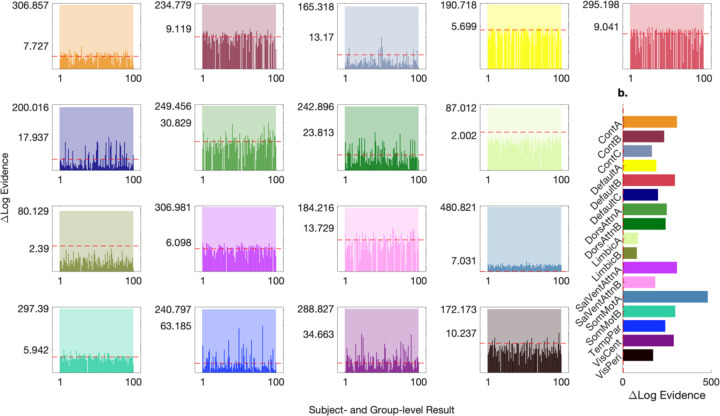
Test-retest reliability of hierarchical empirical Bayes models for the 17 Schaefer brain networks (100 subjects). **a** Bar plots show relative log evidence for hierarchical empirical Bayes models evaluated—both steps 1 and 2—after applying the Bayesian model-average variance transformations identified out of session. Each bar represents the difference in log evidence—at the group (semitransparent bars) and subject (opaque bars) level—between hierarchical empirical Bayes models which utilize empirical constraints—structure-based priors—at the third level, and null (structurally uninformed) models which utilize generic priors at the third level. Note the use of a log-scaled y-axis, with a dashed red line indicating a relative log evidence (or log Bayes factor) of 3, considered strong evidence against the null.38 Tick marks on the y-axis indicate the maximum increase in log evidence at the subject level, and the increase in log evidence at the group level. Log-scaling necessitated the exclusion of several decreases in log evidence at the subject level (see Supplementary Fig. 1 for alternative visualization). **b** Bar plot presents a side-by-side comparison of each relative log evidence for hierarchical empirical Bayes models (the group-level difference in log evidence). Again, a dashed red line indicates a relative log evidence of 3, and tick marks on the y-axis show Schafer network abbreviations (we refer the reader to the network parcellation subsection of the Methods section for network nomenclature). The color scheme for the bars follows a standardized palette and is applied to the plots in the first panel.

Once network-specific BMA variance transformations were identified in the (session 1) exploratory, face-validation dataset, we evaluated their test-retest reliability across time points—on the order of days—with DCMs derived from (session 2) data provided by the same subjects that comprised the exploratory, face-validation dataset. Fig. 3a indicates a consistent pattern in model comparison across the networks, with out-of-session structure-based priors substantially improving the log evidence of the group-level effective connectivity models (note the use of a log-scaled y-axis). For context, Fig. 3b shows that the LimbicB network, compared to the other brain networks, yielded the smallest increase in log evidence for the structurally informed group-level models, as compared to the null (structurally uninformed) group-level models. This increase in log evidence was approximately 80, suggesting there is at least e^80^ ≅ 5 × 10^34^ times more evidence in favour of the model with network-specific structure-based priors identified in session 1, over the null model. Furthermore, Fig. 3a shows that leveraging structurally informed group-level posteriors as empirical priors increased log evidence for most subjects within most networks, above and beyond the increases provided by using the null group-level posteriors as empirical priors.

### Between-subject out-of-sample validation

Next, we assessed the degree to which the network-specific BMA variance transformations identified in the exploratory, face-validation dataset served as a network-specific link between structural connectivity and prior variances over group-level effective connectivity. We conducted two out-of-sample validations (in both validation subjects’ session 1 and session 2 data). This out-of-sample validation mirrored the within-subject reliability test; however, the validation samples’ own structural connectivity data were utilized. Hence, although the network-specific BMA variance transformations remained consistent, the resultant prior variances were inherently different, reflecting the unique (consensus) structural connectivity of the out-of-sample validation sample.

**Fig 4.**
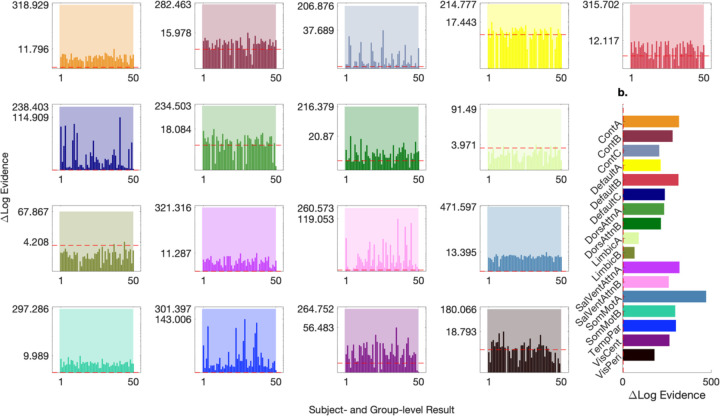
Out-of-sample validation of hierarchical empirical Bayes models for the 17 Schaefer brain networks (50 subjects). **a** Bar plots show relative log evidence for hierarchical empirical Bayes models evaluated—both steps 1 and 2—after applying the Bayesian model-average variance transformations identified out of sample. Each bar represents the difference in log evidence—at the group (semitransparent bars) and subject (opaque bars) level—between hierarchical empirical Bayes models which utilize empirical constraints—structure-based priors—at the third level, and null (structurally uninformed) models which utilize generic priors at the third level. Note the use of a log-scaled y-axis, with a dashed red line indicating a relative log evidence (or log Bayes factor) of 3, considered strong evidence against the null.^38^ Tick marks on the y-axis indicate the maximum increase in log evidence at the subject level, and the increase in log evidence at the group level. Log-scaling necessitated the exclusion of several decreases in log evidence at the subject level (see Supplementary Fig. 2 for alternative visualization). **b** Bar plot presents a side-by-side comparison of each relative log evidence for hierarchical empirical Bayes models (the group-level difference in log evidence). Again, a dashed red line indicates a relative log evidence of 3, and tick marks on the y-axis show Schafer network abbreviations (we refer the reader to the network parcellation subsection of the Methods section for network nomenclature). The color scheme for the bars follows a standardized palette and is applied to the plots in the first panel.

In this section, we present the out-of-sample validation results for session 1 and refer the reader to the Supplementary Figs. 3–4 for the (similar) session 2 results. Fig. 4a indicates that the network-specific variance transformations yielded structure-based priors that, again, substantially improved the log evidence of group-level effective connectivity models. The pattern of increases in log evidence was consistent across the test-retest and out-of-sample validation analyses, with increases highest in one somatomotor (SomMotA) network, and lowest in one limbic (LimbicB) network (see also Supplementary Figs. 3–4). Again, the smallest increase in log evidence for the hierarchical empirical Bayes models was substantial, suggesting there is at least e^67^ ≅ 10^29^ times more evidence in favour of the models with structure-based priors obtained via the application of the network-specific variance transformations, over the null models (Fig. 4b shows this difference in context of other increases in log evidence). Again, for most subjects within most networks, the introduction of these out-of-sample network-specific BMA variance transformations translated to an increase in log evidence at the first level of subject-specific DCMs.

### Inter-network differences in the coupling between structural and effective connectivity

**Fig 5.**
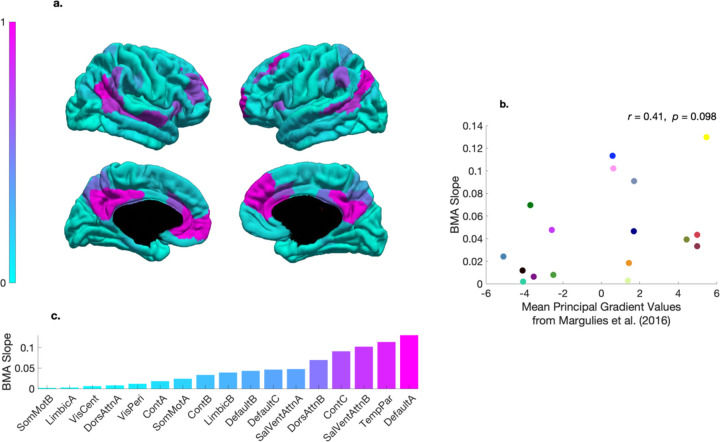
Coupling between structural and effective connectivity across the cortex. **a** Relative weight (or slope) parameters for the network-specific Bayesian model-average (BMA) variance transformations on sagittal views of a cortical pial surface. The colormap indicates that regions in magenta (cyan) have relatively higher (lower) coupling between structural connectivity and effective connectivity. **b** Scatter plot showing the relationship between network-specific BMA slope parameter estimates—we refer the reader to Figs. 1–3 for the corresponding color legend—and the mean principal gradient values per network from ref. ^40^. **c** Bar plot showing that the rank-ordered network-specific BMA beta slope parameter estimates capture key aspects of a well-known cortical (processing) hierarchy. Namely, transmodal (unimodal) networks tend to have been given a relative greater (lesser) weight, with coupling highest in a default mode (DefaultA) network, and lowest in a somatomotor (SomMotB) network.

To examine inter-network differences in the degree to which structural connectivity influenced—i.e., was linearly coupled with—effective connectivity, we compared the weight (or slope) parameters of the BMA variance transformations (i.e., the parameters defining the gradient of the transformations in Fig. 2a). To visualize this measure on a pial surface, each (relative) weight was mapped to a colour from a cyan-magenta gradient colormap (Fig. 5a). Fig. 5c indicates that the relationship between structural connectivity and third-level priors was highest in the default mode (DefaultA) network that comprised hub regions: posterior cingulate cortex (PCC) and medial prefrontal cortex (mPFC). Fig. 5c also shows that these weight parameters were situated along an axis that describes an approximate unimodal (sensory) to transmodal (integrative) processing spectrum. In this way, notwithstanding that these networks have been examined separately, the results recapitulate a key aspect of a previously identified functional hierarchy, which in the human cortex, peaks in regions corresponding to the default mode network, and reaches its nadir in somatomotor regions. Fig. 5b shows a moderate positive (product-moment) correlation (*r* = 0.41) between weight parameters and the mean values from the principal gradient of functional connectivity presented in ref. ^40^, suggesting a possible relationship at the network level. The lack of statistical significance (*p* = 0.098), however, indicates that conclusions regarding this relationship should be drawn with caution.

## Discussion

This study explored whether integrating structural connectivity into a hierarchical model of resting-state effective connectivity improved model evidence. Recently, Sokolov and colleagues introduced a method via which structural connectivity is integrated into a Bayesian random effects model of group-level effective connectivity.^15,18^ Here, we built on this prior work in several ways. First, we incorporated BMA into our procedure to account for uncertainty in the selection of (structural connectivity to) variance transformations. Second, we examined the impact of utilizing structurally informed group-level effective connectivity as empirical priors for re-evaluating subject-level effective connectivity. Third, we applied our hierarchical empirical Bayes procedure to resting-state (rather than task-based) effective connectivity. Fourth, we demonstrated our procedure’s test-retest reliability and out-of-sample validity across resting-state networks, and finally, we examined inter-network differences in the coupling between structural and effective connectivity.

In an exploratory, face-validation phase, the average evidence of group-level effective connectivity models was improved with the introduction of positive, monotonic variance transformations, such that an increase in the strength of a structural connection increased the probability of a (non-zero) effective connection. Although the ContC and DefaultC networks were exceptions to this pattern—with the introduction of structural connectivity-based priors decreasing model evidence on average—we showed, in a within-subjects test-retest reliability phase and a between-subjects out-of-sample validation phase, that the (positive, monotonic) BMA variance transformation derived for all networks demonstrated better generalization and robustness to new data, when compared to structurally uniformed models. Finally, we showed that the coupling between structural and effective connectivity recapitulated a well-described cortical hierarchy,^33–37^ with coupling lowest in unimodal networks (involved in basic sensory processing or motor functions), highest in transmodal networks (involved in complex, integrative processes), and peaking in the DMN.

Our findings provide evidence to suggest that the macroscale structural connectivity of the brain shapes, and improves large-scale models of, directed interactions between neuronal populations, with stronger structural connections increasing the probability of effective connections. This relationship does not appear to be uniform across the brain but rather appears to be modulated along an approximate unimodal-transmodal axis. Importantly, this study provides, to our knowledge, the first demonstration of such a network-dependent modulation of the relationship between structural and effective connectivity in humans (see ref. ^16^ for a related study in mice), demonstrating the predictive validity of the hierarchical empirical Bayes framework, but also hinting at a deeper organizational principle. Namely, that the principal, unimodal-transmodal gradient of functional connectivity may fundamentally be explained in terms of the constraints that structural connectivity exerts on effective connectivity.

Our results are aligned with those from studies that have taken a Bayesian approach to integrating structural and directed connectivity and shown that introducing a positive, monotonic mapping between structural connectivity and the variances of priors over effective or directed functional connectivity parameters, increase model evidence.^13,15,17–19^ More specifically, our work is aligned with prior work examining the impact of structural connectivity-based priors in group-level directed connectivity models.^15,18,19,22^ It differs from this prior work, however, as these investigations have not, per se, investigated the impact of leveraging a structurally informed group-level effective connectivity to constrain subject-level effective connectivity. Furthermore, rather than utilize a sigmoidal variance transformation (first introduced in ref. ^13^), we utilized a linear function amenable to analytic BMA; and, importantly, validated our procedure with new, unseen data.

Our work is similarly related to a) recent mechanistic work that demonstrates that integrating structural connectivity into directed connectivity models can recapitulate known features of brain organisation, in addition to b) ML-based work that demonstrates such integration results in reliable inferences. For example, in Rolls and colleagues’ study, an extended version of the Hopf whole-brain model was used to infer the coupled activity of structurally connected regions in the human visual cortex and recapitulated a hierarchy of interactions observed in the visual cortex of macaques.^24,41^ Also, in a study from Bagheri and colleagues, Bayesian causal discovery frameworks that incorporated structural connectivity data outperformed traditional (structurally uniformed) ML methods vis-à-vis uncovering consistent directed connectivity networks across resting-state fMRI sessions in the Human Connectome Project (HCP) dataset.^26^ Among other differences between studies, the approach employed in the present study allows one to assess the degree of coupling between structural and effective connectivity without a commitment to the idea that (imperfectly measured) structural connectivity provides a deterministic constraint on effective connectivity.

Our findings have important implications. First, they underscore the importance of considering structural connectivity in effective connectivity. Integration of structural connectivity-based priors into hierarchical effective connectivity not only facilitates robust inference, but relative to the structurally uniformed approach that is typically employed in DCM studies,^42,43^ this integration more fully realizes the Bayesian principle of leveraging prior knowledge, and increases the interpretability of priors. In other words, structure-based priors can be understood in relation to a biophysical property of the system being studied. Second, our methodology offers a framework for exploring the brains’ functional integration under pharmacological-based interventions or in psychiatric disorders, where deviations from typical structural-effective connectivity relationships might elucidate (for example) the underlying neuronal mechanisms of disease pathophysiology. Recently, structurally informed directed connectivity models have been used to identify case-control differences in depression, autism, and schizophrenia,^20,44–47^ and may prove useful in disease subtyping. Furthermore, research has suggested that the coupling between structural and functional connectivity decrease under the influence of psychedelics, and it will be important to investigate whether this pattern is evident using the hierarchical empirical Bayes method utilized here.^48^

This study’s implications need to be considered in light of certain limitations. First, although our study builds on the assumption of monotonicity between structural and effective connectivity, supported by prior research, the assumption of linearity and monotonicity may not hold across all neural systems or conditions. Second, the computational challenges inherent in spectral DCM necessitated the independent inversion of resting-state effective connectivity networks, limiting our ability to investigate whole-brain coupling between structural and effective connectivity. Finally, owing to DWI’s inability to determine axonal directionality, our procedure used symmetric structural connectivity. Consequently, in our models, for two given regions, equal priors over effective connections in both directions were assigned. Future research can hope to address these issues by exploring different functional mappings between structural connectivity and effective connectivity, utilizing novel methods that enable inference of whole-brain effective connectivity (e.g., regression DCM),^49^ and exploring principled ways of inferring asymmetric signalling from structural connectivity (see ref. ^50^ for a review of relevant empirical work).

In conclusion, our study further elucidates the role that structural connectivity exerts in shaping effective connectivity. By leveraging a novel two-step hierarchical empirical Bayes method, we reveal that a positive, monotonic relationship between structural connectivity and the prior probability of group-level effective connectivity generalizes out of session and out of sample, increasing model evidence at both group and subject levels. The recapitulation of a unimodal-transmodal cortical hierarchy through inter-network variation in the coupling between structural and effective connectivity highlights the role that structurally constrained lower-order dynamics play in shaping patterns in higher-order statistical dependencies between brain regions. Our findings recommend a shift towards more integrative approaches in DCM-based research, in which the fusion of structural and effective connectivity models could offer novel insights into functional integration in health and disease.

## Methods

### Data

Data used in this study were sourced from the HCP.^51^ Test (face-validation) and retest datasets included data from 100 healthy adults (54 female, age 22–35) acquired on two different days (session 1 and session 2, respectively); and two out-of-sample validation datasets included data from 50 healthy adults (24 female, age 22–35) acquired on two different days.

### Data acquisition and pre-processing

Data comprised both DWI and resting-state fMRI (rfMRI) scans from each participant. DWI data were obtained using a spin-echo echo-planar imaging (EPI) sequence constrained by a repetition time (TR) of 5,520 ms, echo time (TE) of 89.5 ms, a flip angle of 78 degrees, and a multiband factor of 3. The field of view (FOV) was set to 210 mm in the readout (RO) direction and 180 mm in the phase encoding (PE) direction, with a resolution matrix of 168 × 144 (RO × PE). Per participant, the data consisted of 111 slices with a 1.25 mm isotropic voxel size, and diffusion-weighted measurements were performed with b-values of 1,000, 2,000, and 3,000 s/mm2, and six b0 scans were also acquired for signal normalization.

The rfMRI data were acquired using a gradient-echo EPI sequence constrained by a TR of 720 ms, a TE of 33.1 ms, and a flip angle of 52 degrees, and employed a multiband factor of 8. The FOV for rfMRI data was 208 × 180 mm (RO × PE), with a resolution matrix of 104 × 90 (RO × PE). Per participant, the data consisted of 72 slices with an isotropic voxel size of 2 mm. The entire rfMRI scanning session lasted for 14 m 33 s, yielding 1,200 time points of functional brain activity data. The HCP protocol includes data acquired using two phase-encoding directions: left-to-right (L-R) and right-to-left (R-L). For our study, we used the L-R phase direction in each dataset.

The HCP minimally pre-processed pipeline (v3.19.0) was employed for pre-processing the data, with denoising of the functional timeseries data completed using the independent component analysis-based X-noiseifier (FIX) algorithm.^52^ We refer the reader to the extensive documentation of these acquisition and pre-processing steps in ref. ^53^.

### Tractography

Deterministic tractography was performed utilising the fibre assignment by continuous tractography (FACT) algorithm via MRtrix3.^54,55^ First, at each voxel, a single fibre orientation was modelled by the primary eigenvector of a diffusion tensor fitted to DWI data using iterative least squares. Second, 10 million streamlines were propagated voxel-wise, following the direction of the most aligned (colinear) fibre orientations. Streamlines were dynamically seeded from regions in which streamlines were sparse (relative to the fibre density suggested by the underlying fibre orientation distributions),^56^ and either terminated at the gray matter-white matter interface, or at a point where fractional anisotropy (FA) fell below 0.2, curvature exceeded 45 degrees, or length exceeded 250 mm. Finally, these streamlines were downsampled by a factor of 5, and anatomically constrained tractography (ACT) was used to improve their biological plausibility.^57^

### Network parcellation

In our study, rfMRI and tractography data were parcellated into distinct regions of interest (ROIs) using the 200-region cortical atlas developed by Schaefer and colleges.^32^ This atlas, which was generated based on a gradient-weighted Markov Random Field model and resting-state functional connectivity, can be organized according to 17 resting-state networks identified by in ref. ^58^. Each network, on average, consists of approximately 12 distinct regions, which permitted us to effectively investigate network-level dynamics across the cortex while managing the computational complexity associated with inverting large network models.

Tractography data were parcellated using MRtrix3 and a radial search procedure in which streamlines were assigned to parcels within a 5-mm radius of their endpoints. The rfMRI data were parcellated—averaged—using standard HCP Workbench Command tools. Parcellation yielded (200 × 200) structural connectivity matrices, and (200 × 1,200) rfMRI data matrices (capturing time-varying activity across 200 brain regions over 1,200 time points).

With regards to network nomenclature, the 17 networks in Schaefer and colleagues’ atlas include three control networks (ContA, ContB and ContC); three default mode networks (DefaultA, DefaultB and DefaultC); two dorsal attention networks (DorsAttnA and DorsAttnB); two limbic networks (LimbicA and LimbicB); two salience/ventral attention networks (SalVentAttnA and SalVentAttnB); two somatomotor networks (SomMotA and SomMotB); a temporal parietal (TempPar) network; a visual central (VisCent) network; and a visual peripheral (VisPeri) network. It is important to note that these network labels are heuristic in nature: they should be considered as providing a useful but simplified representation of the functional roles that these networks play.^58^

### Structural connectivity

Tractography exhibits well-documented biases whereby a) short-range connections are overrepresented, overshadowing longer-distance tracts; and b) larger brain regions appear disproportionately connected (i.e., connectivity is positively correlated with regional surface area).^59^ To account for these issues, for both the test-retest and validation samples, we generated distance-based consensus structural connectivity matrices per the approach described in ref. ^60^. These matrices retained the distributions of within-hemisphere and between-hemisphere connection lengths (as approximated via the Euclidean distances between parcel centroids). Connections were divided into distance bins, and edges were selected for inclusion in the group matrix based on their consistency across subjects within each bin. The resultant unweighted consensus matrix was multiplied, elementwise, with an average matrix (the mean connectivity values between all pairs of regions across subjects’ data). The resultant weighted consensus matrix was normalized by parcel surface area, yielding a group-level connectome of normalized connection weights.

### Spectral dynamic causal modelling

DCM is a Bayesian framework of inferring effective connectivity, that is, directed interactions between neuronal populations.^10^ It is grounded in the use of a biophysically realistic generative model that simulates how observed data (recorded using fMRI, for example) are generated by dynamic interactions between unobserved neuronal populations.^61,62^ DCM furnishes inferences about the interactions between neuronal populations underlying data through the inversion—the Bayesian fitting—of this generative model. Rather than computing posteriors directly, this inversion is achieved using variational Bayes under the Laplace approximation (i.e., variational Laplace), which involves estimating the mean and covariance—the sufficient statistics—of Gaussian posterior densities for model parameters in a manner that seeks to optimize the trade-off between the accuracy of data fit and the complexity of the model.^63^ This balance is captured by the free energy, a quantity that approximates the (log) model evidence, and thereby facilitates model comparison.^39^

Spectral DCM,^64^ a variant of DCM applied to rfMRI in the spectral (or frequency) domain, has been rigorously validated through in silico simulations, optogenetic studies in rodents, and assessments of reliability.^65–67^ In our study, for each of the 150 subjects, across 17 networks and two sessions, a spectral DCM was specified and inverted using the variational Laplace method as implemented in the statistical parametric mapping toolbox (SPM12). The generative model utilized in spectral DCM is a continuous-time state-space model that describes how latent neuronal activity gives rise to observed BOLD signals, y:

(1) $$\dot{\text{x}}(t)=Ax(t)+v(t)\;\;\;\;\;\;\;\;\;\;\;\;\;\;\;(state\;equation)\;y(t)=h(t)*x(t)+e(t)\;\;\;\;\;\;\;\;\;(observation\;equation)$$

In a model with n neuronal populations, $x(t)={\left[x_{1}(t),\ldots ,x_{n}(t)\right]}^{T}$ represents the unobserved, or latent, state of neuronal populations at time t, $h\left(t\right)$ signifies the parameterized hemodynamic response. The matrix $A\in {\mathbb{R}}^{n\times n}$ encodes the rate of intra- and inter-regional effective connectivity, in its diagonal and off-diagonal elements, respectively, with the former representing the intrinsic excitability of each region. In spectral DCM, both v(t) and e(t) are parameterized as power-law noise. Using the Fourier transform, F, the state-space model is projected into the frequency domain, generating predictions about the cross-spectral density of the signal y(t) at frequency $\omega :G_{y}(\omega )=\left[F\{y(t)\}F{\left\{y(t)\right\}}^{†}\right]$. Here, † represents the conjugate transpose. The full model reads:

(2) $$G_{y}(\omega )=H(\omega ){\left(i\omega I-A\right)}^{-1}G_{v}(\omega ){\left(-i\omega I-A^{T}\right)}^{-1}H{\left(\omega \right)}^{†}+G_{e}(\omega ),$$

where H(ω) is the Fourier transform of the hemodynamic response function, I is an n-dimensional identify matrix, and $G_{v}(\omega )$ and $G_{e}(\omega )$ are the cross-spectral density of v(t) and e(t), at frequency ω, respectively. Importantly, the latent neuronal state in the frequency domain, X(ω), has been factored out of the equations via the substitution $G_{v}(\omega )=(i\omega I-A)\;X(\omega )X{\left(\omega \right)}^{†}\left(i\omega I-A^{T}\right)$. We refer the reader to ref. ^68^ for a didactic introduction to these derivations.

In this study, in contrast to other work examining structurally informed directed connectivity, DCMs were configured such that all possible effective connections—i.e., all entries in the transition matrix–could be inferred, regardless of whether they were supported by a corresponding structural connection. All model parameters were equipped with priors, and importantly for this study, before inversion, the variance of zero-mean Gaussian priors over all interregional effective connections was set to 1/2, while the prior variance of all intraregional effective connections was set to 1/64.

### Hierarchical empirical Bayes procedure

After estimating individual DCMs, researchers often aim to quantify group differences in effective connectivity or hemodynamic responses to test hypotheses about the effect of, for example, an intervention or disease. One approach to estimating these group differences, embedded in the parametric empirical Bayes (PEB) framework,^42^ involves treating the subject-level posteriors over parameters of interest as a Bayesian general linear model (GLM) that incorporates second-, group-level parameters as fixed effects, and random effects that encode sources of inter-subject variation. In the absence of a specific hypothesis about inter-subject variation—that one could encode in a design matrix—a PEB model in which effective connectivity is treated as a fixed effect, reads:

(3) $$A^{\left(2\right)}=p\left(A^{\left(2\right)}\right)+\varepsilon^{\left(3\right)}\;\;\;\;\;\;\;\;\;\;\;\;\;\;\;(third-levelmodel)\;A_{i}^{\left(1\right)}=\Gamma^{\left(2\right)}\left(A^{\left(2\right)}\right)+\varepsilon^{\left(2\right)}\;\;\;\;\;\;\;\;\;\;\text{(}second-levelmodel\text{)}\;y_{i}=\Gamma_{i}^{\left(1\right)}\left(A_{i}^{\left(1\right)}\right)+\varepsilon_{i}^{\left(1\right)}\;\;\;\;\;\;\;\;\;\;\;\;\;\;(first-levelmodel)$$

Here, subject-level effective connectivity, $A_{i}^{\left(1\right)}$, encoded in individual DCMs, $\Gamma_{i}^{\left(1\right)}$, is modelled via a Bayesian GLM, $\Gamma^{\left(2\right)}$, comprising second-, group-level effective connectivity $A^{\left(2\right)}$, and zero-mean (i.i.d.) additive noise, $\varepsilon^{\left(2\right)}$, which constitutes a between-subject random effect. The first- and third-level error terms— $\varepsilon_{i}^{\left(1\right)}$, $\varepsilon^{\left(3\right)}$—represent residuals. Importantly, group-level effective connectivity is equipped with priors $p\left(A^{\left(2\right)}\right)$, at the third level, enabling the framework to furnish group-level posteriors. These group-level posteriors can then be utilized as empirical priors that constrain the re-inversion of subject-level parameters. In practice, however, PEB proceeds in two steps: the group-level (Bayesian GLM) model is inverted using variational Laplace, and the subject-level posteriors are re-evaluated analytically using BMR, without explicit re-inversion.

BMR exploits the fact that if two Bayesian models differ only in their priors, the posteriors and evidence of a reduced model can be computed, analytically, from the posteriors and evidence of a full (parent) model (under the assumption that the likelihood remains the same for all the models).^69–72^ If one considers subject-level effective connectivity (in equation 3), for example, the evidence for a reduced model, relative to a full model $\frac{p\left(y_{i}|\Gamma_{i}^{\prime (1)}\right)}{p\left(y_{i}|\Gamma_{i}^{\left(1\right)}\right)}$ (or Bayes factor), and posteriors for a reduced model $p\left(A_{i}^{\left(1\right)}|y_{i},\Gamma_{i}^{\prime (1)}\right)$, is obtained via the following:

(4) $$\frac{p\left(y_{i}|\Gamma_{i}^{\left(1\right)}\right)}{p\left(y_{i}|\Gamma_{i}^{\left(1\right)}\right)}=\int p\left(A_{i}^{\left(1\right)}|y_{i},\Gamma_{i}^{\left(1\right)}\right)\frac{p\left(A_{i}^{\left(1\right)}|\Gamma_{i}^{\left(1\right)}\right)}{p\left(A_{i}^{\left(1\right)}|r_{i}^{\left(1\right)}\right)}dA_{i}^{\left(1\right)}\;\;\;\;\;\;\;\;\;\;\;\;\;\;\;\;\;(relative\;evidence)\;p\left(A_{i}^{\left(1\right)}|y_{i},\Gamma_{i}^{\prime (1)}\right)=p\left(A_{i}^{\left(1\right)}|y_{i},\Gamma_{i}^{\left(1\right)}\right)\frac{p\left(A_{i}^{\left(1\right)}|\Gamma_{i}^{\prime (1)}\right)}{p\left(A_{i}^{\left(1\right)}|\Gamma_{i}^{\left(1\right)}\right)}\frac{p\left(y_{i}|\Gamma_{i}^{\left(1\right)}\right)}{p\left(y_{i}|\Gamma_{i}^{\prime (1)}\right)}\;\text{ (}reducedposteriors\text{)}$$

In this formulation, priors for the parameters of the reduced (full) model are situated in the numerator (denominator) of the second, right-hand terms of both equations. Under the variational Laplace framework, in which priors and posteriors are approximated via the sufficient statistics of Gaussian densities, and evidence is approximated via free energy, an (approximate) analytic solution for this formulation becomes available. For comprehensive mathematical details, we direct the reader to ref. ^42^. In alignment with previous work,^15,18,19^ we treated effective connectivity as a fixed effect in PEB models—a simple, second-level model of the form $A_{i}^{\left(1\right)}=A^{\left(2\right)}+{\text{ε}}^{\left(2\right)}\;$—and assigned empirical priors based on auxiliary data—structural connectivity—to group-level effective connectivity parameters. In so doing, we nested one empirical Bayes procedure within another—incorporating data at multiple hierarchical levels (Fig. 1). Hence, we refer to the procedure as a hierarchical empirical Bayes procedure.

The translation of structural connectivity to third-level priors necessitated a suitable transformation model. Consistent with prior work that has identified a monotonic relationship between structural and directed connectivity—with the strength of structural connectivity scaling the probability of directed connectivity^13,15,18,19^—we used a linear transformation of normalized structural connectivity to the variances of zero-mean Gaussian priors over group-level effective connectivity, where increased variance corresponds to increased probability of (non-zero) effective connectivity. Mathematically, this transformation is represented as:

(5) $$\varphi_{i,j}^{\prime }=\frac{\varphi_{i,j}}{\max\;(\varphi )}\;\;\;\;\;\;\;\;\;\;\;\;\;\;\;\;\;\;\;\;\;\;\;\;\;\;\;\;\;\;\text{ (}normalizedstructuralconnectivity\text{)}\;\sigma_{i,j}^{2}=\beta \varphi_{i,j}^{\prime }+\alpha \;\;\;\;\;\;\;\;\;\;\;\;\;\;\;\;\;\;\;\;\;\;\;\;\;\;\;\;\;\;(variancetransformation)\;p\left(A_{i,j}^{\left(2\right)}|m\right)=N\left(A_{i,j}^{\left(2\right)};0,\sigma_{i,j}^{2}\right)\;\;\;\;\;\;\;\;(third-levelpriors)$$

Here, φ is an n×n symmetric matrix encoding the structural connectivity for the relevant n-region network. The normalized structural connectivity weights, $\varphi_{i,j}^{\prime }$, are transformed into variance terms, $\sigma_{i,j}^{2}$, for third-level priors over second-, group-level interregional effective connectivity $A_{i,j}^{\left(2\right)}$, where i≠j. The parameters α and β determine the intercept and slope (weight) of the variance transformation, respectively.

### Face-validation, test-retest, and out-of-sample validation phases

When seeking to implement this hierarchical empirical Bayes procedure, one can take at least two approaches. First, per the exploratory, face-validation phase of this study, one can examine the influence of third-level empirical priors post hoc, after the inversion of the second-, group-level model. Second, per the within-subjects test-retest and between-subjects out-of-sample validation phases, third-level empirical priors can be pre-specified ahead of model inversion. This approach ensures that an a priori parameterization of the variance transformation influences results from the outset. These approaches are elaborated in the following paragraphs.

In the exploratory, face-validation phase, per network, we first used the test (face-validation) dataset DCMs to invert a (parent) group-level model using broad, generic third-level priors: the priors over interregional effective connectivity had a variance of 1/2, while priors over intraregional effective connectivity had a variance of 1/64. We then used BMR to evaluate the (relative) model evidence associated with different reduced models, varying the parametrization of the variance transformation. In these analyses, 𝛼 ranged from −1/2 to 1/2, and β ranged from 0 to 1/2, with both parameters sampled at 30 equidistant points. To ensure numerical stability and the fulfilment of assumptions—in the context of BMR, alternative models are assumed to include priors that are more precise than those of a parent model (i.e., $\sigma_{i,j}^{2}\leq 1/2$)—parameter combinations for which α+β exceeded the range [1 × 10^−5^, 1/2] were omitted (resulting in the assessment of 7,273 unique structural connectivity-to-variance relationships).

Having assessed the relative log evidence associated with each parametrization of the variance transformation per network, we proceeded to assess the reliability of the BMA variance transformation for each network in a within-subjects test phase. Here, the BMA variance transformation is the evidence-weighted average of the (linear) variance transformations, given by:

(6) $$V_{BMA}=\frac{\sum_{k}e^{F\left(m_{k}\right)}\left(\beta_{k}\cdot x+\alpha_{k}\right)}{\sum_{k}e^{F\left(m_{k}\right)}},$$

where, $F\left(m_{k}\right)$ is the relative log evidence for the k-th alternative model, $\alpha_{k}$ and $\beta_{k}$ are parameters for the k-th alternative model. We used the network-specific BMA variance transformation to specify the third-level structure-based priors of for hierarchical empirical Bayes models of DCMs fitted to the retest dataset. These structurally informed models were then inverted, and compared to equivalent PEB models that incorporated the same DCMs yet lacked third-level structure-based priors (i.e., these null models utilized the broad, generic third-level priors described in relation to the exploratory, face-validation phase).

To further assess the robustness of the network-specific BMA variance transformations, we conducted two out-of-sample validations within an independent sample, across two sessions. This out-of-sample validation mirrored the reliability analysis; however, the validation samples’ own structural connectivity data were utilized. Hence, although the variance transformation remained consistent, the resultant prior variances were inherently different, reflecting the distinct (consensus) structural connectivity of the validation sample.

### Cortical gradient of functional connectivity

Margulies and colleagues applied a diffusion embedding algorithm to rfMRI data to extract latent components, referred to as gradients, underlying functional connectivity.^40^ The principal gradient separated unimodal and transmodal regions along an approximate sensory-fugal axis,^33^ with somatomotor and DMN regions situated at opposite ends. Leveraging this principal gradient as a quantitative description of this spectrum, we downsampled the gradient from vertex to network resolution by averaging gradient values per each of the 17 Schaefer networks and compared these mean principal gradient values to the weight parameter estimates of the network-specific BMA variance transformations.

## Supplementary Material

Supplement 1

## Data Availability

This study's analyses were conducted using datasets that are publicly accessible. The mapping of structural and effective connectivity networks in the human brain was performed using data from the Human Connectome Project, available at: https://db.humanconnectome.org/. Shaefer and colleagues’ brain parcellation—see ref. ^32^—is accessible here: https://github.com/ThomasYeoLab/CBIG/. The cortical gradient of functional connectivity, associated with ref. ^40^, can be found at: https://www.neuroconnlab.org/data/index.html.

## Footnotes

† In this text, DCM refers both to the dynamic causal modelling framework, and an instantiation of a dynamic causal model.

‡ In this text, BMA refers both to the Bayesian model average of various mathematical objects (e.g., Bayesian model-average function), and to the process of Bayesian model averaging itself.
