# Structurally informed resting-state effective connectivity recapitulates cortical hierarchy

**DOI:** 10.1101/2024.04.03.587831

**Published:** 2025-02-28

**Authors:** Matthew D. Greaves, Leonardo Novelli, Adeel Razi

**Affiliations:** 1 School of Psychological Sciences, Monash University, Clayton, Victoria, Australia; 2 Monash Biomedical Imaging, Monash University, Clayton, Victoria, Australia; 3 Wellcome Centre for Human Neuroimaging, University College London, London, UK; 4 CIFAR Azrieli Global Scholars Program, CIFAR, Toronto, Ontario, Canada

## Abstract

Neuronal communication relies on the anatomy of the brain, yet it remains unclear whether, at the macroscale, structural (or anatomical) connectivity provides useful constraints on effective connectivity. Here, we assess a hierarchical empirical Bayes model that builds on a well-established dynamic causal model by integrating structural connectivity into resting-state effective connectivity via priors. *In silico* analyses show that the model successfully recovers ground-truth effective connectivity and compares favorably with a prominent alternative. Analyses of empirical data reveal that a positive, monotonic relationship between structural connectivity and the prior probability of group-level effective connectivity generalizes across sessions and samples. Finally, attesting to the model’s biological plausibility, we show that inter-network differences in the coupling between structural and effective connectivity recapitulate a well-known unimodal-transmodal hierarchy. These findings underscore the value of integrating structural and effective connectivity to enhance the understanding of functional integration in health and disease.

## Significance statement

To advance the understanding of how neuronal populations interact *in vivo*, it is essential to develop models that integrate neuroimaging modalities. Here, we show how dynamic causal modeling of resting-state effective connectivity is substantially improved via the integration of structural connectivity data, yielding reliable, valid inferences. This integration reveals that structural connectivity’s influence on effective connectivity varies along an established unimodal-transmodal cortical hierarchy. This finding provides the first evidence of a network-dependent modulation of the relationship between structural and effective connectivity in humans. Against the backdrop of sustained and widespread interest in dynamic causal modeling, this study suggests that without integrating structural connectivity-based constraints, current applications of dynamic causal modeling risk overlooking significant insights into brain dynamics.

## Introduction

Computational modeling facilitates the mapping of both structural and effective connectivity in humans using *in vivo* magnetic resonance imaging (MRI)^1,2^. Structural (or anatomical) connectivity refers to a network of nerve tracts (bundles of axons), while effective connectivity refers to the directed, time-dependent flow of information between neuronal populations. Although it is reasonable to assume that neuronal populations primarily communicate via prominent nerve tracts, understanding how structural connectivity constrains effective connectivity remains an open challenge^3^.

Here, we examine a hierarchical empirical Bayes model in which structural connectivity constrains both group- and subject-level resting-state (task-free) effective connectivity. At the first (subject) level, we utilize a well-established biophysical model—a dynamic causal model (DCM)^4,5^—that describes how directed interactions between a network of unobserved neuronal populations cause changes in blood-oxygen-level-dependent (BOLD) signals obtained via functional MRI (fMRI). By generating synthetic data that replicates empirical data, this DCM facilitates—via model inversion (parameter estimation by fitting to observed data)—a move backwards from effects (observed data) to underlying causes (interregional neuronal communication, or effective connectivity). Given that this problem of determining underlying causes is ill-posed (many parameter configurations can explain the observed data equally well), model inversion via Bayesian methods—in which uncertainty about model parameters is represented by probability distributions—is often preferred over methods that seek explicit solutions^6^.

**Fig. 1.**
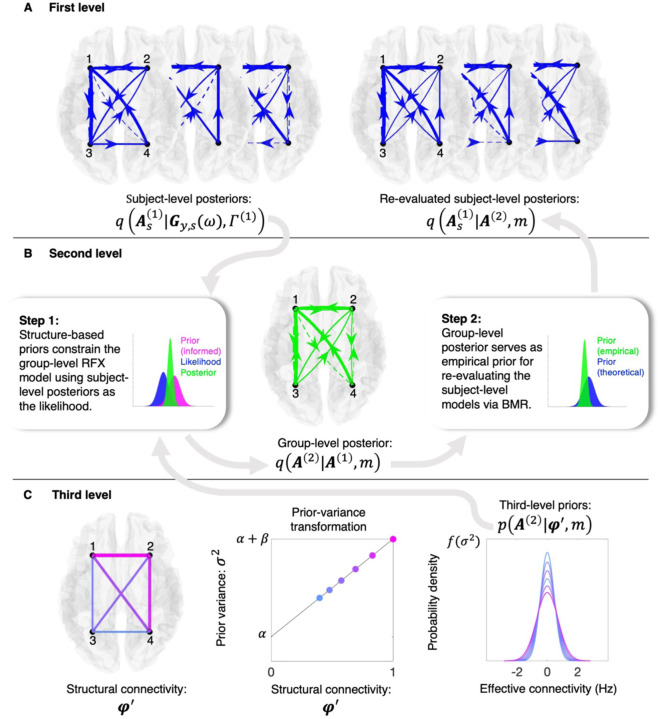
Hierarchical empirical Bayes framework for structurally informed effective connectivity. A three-level hierarchical model for inferring effective connectivity using empirical priors and Bayesian model reduction (BMR). Arrows and inserts depict information flow and probability notation is consistent with that utilized in the Methods section. Connectivity graphs are formatted such that solid (dashed) lines represent positive (negative) connections, with line weight reflecting connection strength. Color-coded probability density functions (PDFs) within inserts indicate the level at which a given density originates—first (blue), second (green), and third (magenta)—and show a group-level posterior (Step 1; first insert) serving as an empirical prior (Step 2; second insert). (**A**) A dynamic causal model, $\Gamma^{\left(1\right)}$, is used to infer the (approximate) posterior distributions $q\left(\cdot \right)$ over subjects’ effective connectivity parameters, ${\text{A}}_{s}^{\left(1\right)}$, for subjects s=1,…,S, given their data (${\text{G}}_{y,s}\left(\omega \right)$; left panel). Posteriors are incorporated into the hierarchical model m, and re-evaluated in view of inferred group-level effective connectivity (${\text{A}}^{\left(2\right)}$; right panel). (**B**) Structural connectivity-based priors (structure-based priors) constrain the inversion of a group-level model that utilizes subject-level posteriors as a likelihood (Step 1; first insert). This first step utilizes variational Bayes under the Laplace approximation. The inferred group-level posterior is then used to re-evaluate subject-level models via BMR-based comparison with a theoretical (or non-informed) prior (Step 2; second insert). Here, ${\text{A}}^{\left(1\right)}$ represents the concatenated set of subjects’ effective connectivity parameters, ${\text{A}}_{s}^{\left(1\right)}$. (**C**) The third level model furnishes structure-based priors based on normalized structural connectivity (${\text{φ}}^{\prime }$; first panel). Note that the structural connectivity graph is undirected, with both line weight and color (from a gradient colormap) reflecting connection strength. Normalized structural connectivity is mapped linearly to variances of zero-mean Gaussian priors ($\sigma^{2}$; second panel). Y-axis tick marks indicate the transformation evaluated at the lower $\left(\alpha \right)$ and upper $\left(\alpha +\beta \right)$ bounds of the input parameter space. Finally, third-level priors ($p\left({\text{A}}^{\left(2\right)}|{\text{φ}}^{\prime },m\right)$; third panel) are shown as PDFs, with colors related to the connections and prior variances in the first and second panels, respectively.

The Bayesian model inversion of DCMs involves updating a prior distribution (or priors) for effective connectivity parameters based on observed data, yielding a posterior distribution (or posteriors) for the parameters (Fig. 1A). In the hierarchical empirical Bayes model considered here, these first-, subject-level posteriors are utilized in a second-level analysis, assuming they are a random sample drawn from a group-level (or population) distribution (Fig. 1B). This assumption is modeled with a simple random effects (RFX) model that captures group-level effective connectivity and inter-subject variability^7^. An important aspect of this approach is that the group-level effective connectivity parameters of this RFX model are equipped with priors that, in a third-level model, are informed by structural connectivity obtained via tractography applied to diffusion-weighted MRI (dwMRI) data (Fig. 1C)^8,9^. In simple terms, a linear transformation of normalized structural connectivity scales the noisiness of group-level effective connectivity estimates.

In this way, the integration of structural connectivity-based priors (henceforth referred to as structure-based priors) serves to constrain the model inversion of group-level effective connectivity, yielding group-level posteriors that can serve as empirical priors (estimated from the data) for re-evaluating first-, subject-level effective connectivity. Using *in silico* analyses, we show that the model successfully recovers ground-truth effective connectivity and compares favorably with a prominent structurally informed multivariate autoregressive (MVAR) model^10^. In exploratory analyses, we find—in line with theoretical expectations—a positive, monotonic relationship between structural connectivity and (the prior probability of non-zero) effective connectivity across 17 different resting-state networks associated with Schaefer and colleagues’ brain atlas^11^. In tests of reliability and validity, we establish that network-specific relationships between structural and effective connectivity generalize out of session and out of sample. Finally, attesting to the model’s biological plausibility and relevance, we explore inter-network differences in the coupling between structural and effective connectivity and show that these differences align with an established cortical hierarchy as quantified by the principal gradient of functional connectivity^12^.

## Results

Inferring effective connectivity from data using the hierarchical empirical Bayes model (introduced formally in the Methods section) involves updating priors, where the *a posteriori* expectation and uncertainty of a model’s parameters is determined by combining priors with a likelihood function (that describes how well the model’s output fits the data). Formally, this process relies on Bayes’ rule:

(1) $$p\left(\text{θ}|\text{y}\text{,}m\right)=\frac{p\left(\text{y}|\text{θ}\text{,}m\right)p\left(\text{θ}|m\right)}{p\left(\text{y}|m\right)},$$

where $p\left(\text{θ}|\text{y}\text{,}m\right)$ is the posterior probability of model parameters, θ, given the observed data, y, and the model, m. On the right-hand side, $p\left(\text{y}|\text{θ}\text{,}m\right)$ is the likelihood of the data being observed given the model and its parameters, and $p\left(\text{θ}|m\right)$ is the prior probability of the parameters given the model. These priors, $p\left(\text{θ}|m\right)$, can be informative (for example, in this context, the prior probability of non-zero effective connectivity is assumed to scale with a linear transformation of normalized structural connectivity; Fig. 1C) or non-informative if little prior knowledge exists. So-called empirical priors are those which have been derived from the data itself (for example, when second-level models are used to re-evaluate first-level parameter estimates in a hierarchical framework; second insert, Fig. 1B).

Calculating the model evidence, $p\left(\text{y}|m\right)$, which represents the overall probability of the data being observed under the model, requires integrating (or marginalizing) the likelihood over all possible values of the parameters, weighted by the prior distribution. In practice, computing this quantity is often infeasible, and thus, in this context, we make use of the well-known variational Bayes method under the Laplace approximation (VBL), which assumes that the true posterior distribution is Gaussian around its mode^6,13^. Assuming Gaussian prior and likelihood, the fundamental VBL equation is:

(2) $$\text{ln}\;p\left(\text{y}|m\right)\approx F_{m}=E_{q\left(\text{θ}|m\right)}\left[\text{ln}\;p\left(\text{y}|\text{θ}\text{,}m\right)\right]-\text{KL}\left(q\left(\text{θ}|m\right)∥p\left(\text{θ}|m\right)\right).$$

Here, $F_{m}$, is the (variational) free energy—an approximation of the log-model evidence—for the model, m, $E_{q\left(\text{θ}|m\right)}\left[\text{ln}\;p\left(\text{y}|\text{θ}\text{,}m\right)\right]$ is the expected log-likelihood under a variational—approximate posterior—distribution, $q\left(\text{θ}|m\right)$, and $\text{KL}\left(q\left(\text{θ}|m\right)∥p\left(\text{θ}|m\right)\right)$ is the Kullback-Leibler divergence between the variational distribution, $q\left(\text{θ}|m\right)$, and prior distribution. This formulation permits the optimization of $q\left(\text{θ}|m\right)$ such that free energy is maximized, and the model optimally balances accuracy and complexity^6^. For an explanation of how the free energy is derived from the sufficient statistics—mean $\left(\text{μ}\right)$ and covariance $\left(\sum \right)$ —of the densities in Equation 2, we refer the reader to the supporting information (SI Methods).

Several of the results we present in this section reflect assessment of whether structure-based priors yield more parsimonious models (those that better balance accuracy and complexity). Conveniently, the free energy facilitates straightforward model comparison, where the log-Bayes factor is the difference between the log-model evidence assigned to a given set of observations under competing models. This permits comparison of alternative (inverted) hierarchical empirical Bayes models that differ, for example, in terms of whether a model, $m_{1}$ incorporates structure-based priors, or whether the model, $m_{2}$, incorporates non-informative priors:

(3) $$\text{ln}\;{\text{BF}}_{\text{1,2}}=\text{ln}\;p\left(\text{y}|m_{1}\right)-\text{ln}\;p\left(\text{y}|m_{2}\right)\approx F_{m1}-F_{m2}.$$

A Positive log-Bayes factor, lnBF, indicates that the data provide more support for model $m_{1}$ over $m_{2}$, with larger values representing stronger evidence. A log-Bayes factor of 3 or higher is often considered strong— $e^{3}\approx 20$ times more—evidence in favor of $m_{1}$ ^14^.

For efficiency, we take an analytic approach to computing the approximate posterior and free energy for hierarchical empirical Bayes models with structure-based priors from a model with non-informative priors^9^. This analytic procedure, termed Bayesian model reduction (BMR)^7^, is made possible due to the Gaussian form of Equation 2. BMR enables one to adjust the adjust the sufficient statistics of the prior, $p\left(\text{θ}|m_{R}\right)$, and obtain the free energy, $F_{m,R}$, and posterior, $q\left(\text{θ}|m_{R}\right)$, for a reduced (alternative) model, $m_{R}$, from a full (parent) model, m, analytically (without the need for optimization). Specifically, BMR yields the reduced model via the following relationships:

(4) $$q\left(\text{θ}|m_{R}\right)\approx q\left(\text{θ}|m\right)\frac{p\left(\text{θ}|m_{R}\right)}{p\left(\text{θ}|m\right)}\;\;\;\;\;\;\;\;\;\;\;\;\;\;\;\;\left(reduced\;posterior\right)\;F_{m,R}\approx \ln\int q\left(\text{θ}|m\right)\frac{p\left(\text{θ}|m_{R}\right)}{p\left(\text{θ}|m\right)}d\text{θ}+F_{m}\;\;\;\left(reduced\;free\;energy\right)$$

where, per this study, the reduced prior might depend on structural connectivity (Fig. 1C). Note that the likelihood terms do not appear in these expressions, as we assume the full and reduced models have identical likelihoods (allowing them to cancel out). For a more detailed illustration of BMR with Gaussian densities, we refer the reader to the supporting information (SI Methods).

### Face and construct validity

#### In silico parameter recovery

In an *in silico* assessment of face validity, we generated 50 instantiations of BOLD-like time-series data for a 6-region effective connectivity network (see SI Methods). In these simulations, group-level effective connectivity was derived from a standard Gaussian noise process scaled by a linear transformation of normalized structural connectivity, and subject-level effective connectivity was modeled as random deviations from group-level effective connectivity.

Inference proceeded in a multi-step fashion. First, the hierarchical model was inverted under non-informative priors (Steps 1 and 2; Fig. 1). Then, focusing on the second-, group-level RFX model, we utilized a grid search and BMR to evaluate different parametrizations of the structural-connectivity-to-prior-variance transformation (henceforth referred to as the prior-variance transformation; second panel, Fig. 1C) and score the resultant (structurally informed) reduced models against the (non-informed) full model in terms of the log-Bayes factor. This enabled the derivation of a Bayesian model-average (BMA) prior-variance transformation, where each transformation was weighted according to its normalized Bayes factor (see Procedures). Finally, using BMR, the (non-informed) group-level model was re-evaluated under structure-based priors furnished via the BMA prior-variance transformation, and the posterior for the re-evaluated (structurally informed) group-level model was then treated as the prior under which the (non-informed) subject-level models were re-evaluated using BMR (right panel; Fig. 1A).

**Fig. 2.**
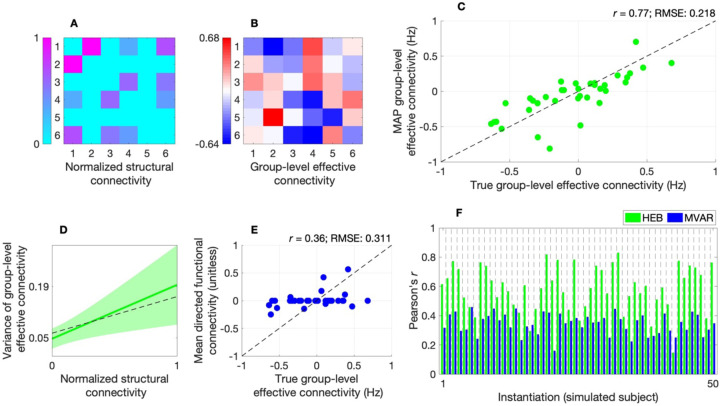
*In silico* evaluation of hierarchical empirical Bayes model. (**A**) Heatmap of normalized structural connectivity utilized in simulations. (**B**) Heatmap of group-level effective connectivity (derived from normalized structural connectivity) utilized in simulations. (**C**) Parity plot for the hierarchical empirical Bayes (HEB) model, showing maximum a posteriori (MAP) estimates of group-level effective connectivity plotted against true values. Scatter points are clustered around the (dashed) identity line with a Pearson’s (product-moment) correlation $\left(r\right)$ of 0.77, and root mean squared error (RMSE) of 0.218, indicating acceptable model performance. (**D**) Bayesian model-average (BMA) prior-variance transformation (solid green line) with shaded area indicating standard error. The BMA transformation closely approximates the ground-truth variance of group-level effective connectivity (dashed black line). Y-axis tick marks indicate the BMA transformation evaluated at the lower and upper bounds of the input parameter space. (**E**) Parity plot for the multivariate autoregressive (MVAR) model showing a lower correlation, higher RMSE, and increased zero-valued estimates. The mean directed functional connectivity is quantified via the mean of (unitless) autoregressive parameters across subject-level models. (**F**) Bar plot of correlation between true and estimated subject-level effective connectivity characterized via the HEB model (green bars) and MVAR model (blue bars) across 50 instantiations, highlighting the greater performance of the former model.

Fig. 2A and Fig. 2B show the normalized (sparse) structural connectivity network and group-level effective connectivity network utilized in this analysis, respectively. The parity plot (Fig. 2C) for the hierarchical empirical Bayes model indicates that—under a signal-to-noise ratio of 1—the model achieves acceptable accuracy, with maximum a posteriori (MAP) estimates of group-level effective connectivity closely distributed around the identity line, a Pearson’s (product-moment) correlation $\left(r\right)$ of 0.77, and root mean squared error (RMSE) of 0.218. Furthermore, Fig. 2D indicates that the BMA prior-variance transformation closely approximates the ground-truth variance of group-level effective connectivity.

To provide construct validation, we show that the model outperforms a prominent structurally informed multivariate autoregressive (MVAR) model of directed functional connectivity, for which structural connectivity serves as a template for possible interactions (such that directed functional connectivity is estimated from region i to region j if a direct structural connection exists between the two regions^10^). Relative to the hierarchical empirical Bayes model, this MVAR model showed lower accuracy at the group level (Fig. 2E) and tended to show poorer ability to recover effective connectivity at the subject level (Fig. 2F; see SI Results, Fig. S1A–B, for additional error metrics). Finally, a macro F1-score demonstrates the superior ability of the hierarchical empirical Bayes model to classify positive, negative, and absent group-level effective connections across a range of noise conditions (SI Results, Fig. S1C).

#### Empirical parameter exploration

In an empirical face-validation phase we utilized both resting-state fMRI data—the session-1 recordings—and structural connectivity (obtained via tractography applied dwMRI data^15–18^) from 100 healthy adults (54 female, age 22–35) sourced from the human connectome project (HCP)^19^. For each brain network, we inverted a separate hierarchical empirical Bayes model following procedures utilized in the preceding *in silico* analyses (see Procedures section). This enabled the derivation of a BMA prior-variance transformation per network.

**Fig. 3.**
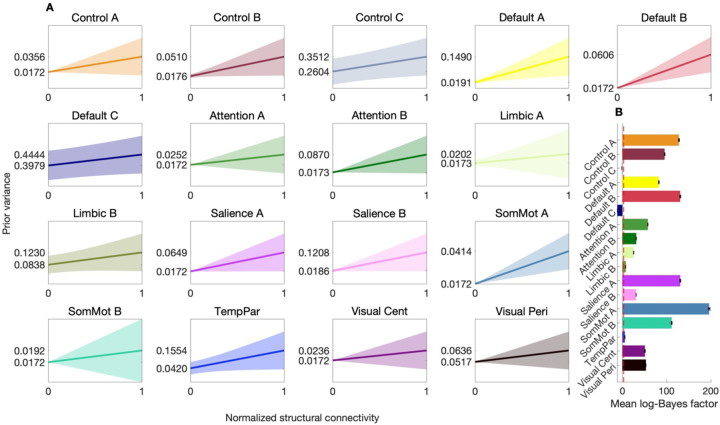
Bayesian model-average prior-variance transformation for 17 brain networks. (**A**) Each panel represents results for a hierarchical empirical Bayes model of a distinct effective connectivity brain network showing the Bayesian model-average (BMA) prior-variance transformation (solid line) as a function of normalized structural connectivity. The shaded regions indicate the standard error (SE) envelope, reflecting uncertainty in the gradient of the BMA functions. Y-axis tick marks indicate the BMA transformation evaluated at the lower and upper bounds of the input parameter space. Networks analyzed include control (Control A–C), default mode (Default A–C), attention (Attention A, B), limbic (Limbic A, B), salience (Salience A, B), somatomotor (SomMot A, B), temporoparietal (TempPar), and visual networks (Visual Cent, central; Visual Peri, peripheral). (**B**) Mean log-Bayes factors for each network with error bars showing the SE. The introduction of structure-based priors consistently increased model evidence, except for one control (Control C) and one default mode (Default C) network. Note a dashed red line indicates log-Bayes factor of 3.

Fig. 3A illustrates that, despite the evaluation of parameter regimes resulting in flat prior-variance transformations, the BMA prior-variance transformation for each network was a positive, monotonic function. Thus, models in which structural connectivity positively scaled with the prior probability of non-zero group-level effective connectivity were, on average, the most parsimonious. The standard error envelopes shown in Fig. 3A illustrate some uncertainty regarding the gradient of these BMA functions and motivated the need to evaluate their reliability and out-of-sample validity. As depicted in Fig. 3B, the mean log-Bayes factors demonstrated that, with the exception of one control (Control C) and one default mode (Default C) network, the inclusion of structure-based priors substantially increased model evidence.

### Test-retest reliability

Once network-specific BMA prior-variance transformations were identified using the session-1 dataset, we evaluated their test-retest reliability across time points—on the order of days—with hierarchical empirical Bayes models inverted using the session-2 data provided by the same subjects (see Procedures). Specifically, we compared hierarchical empirical Bayes models with non-informative priors to hierarchical empirical Bayes models with structure-based priors furnished by applying the relevant, network-specific BMA prior-variance transformation (Fig. 3A). Results (SI Results, Figs. S2–S3) indicate a consistent pattern across the networks, with substantially greater evidence for the models with out-of-session structure-based priors, and all MAP estimates of effective connectivity were in a plausible range.

### Out-of-sample validity

Next, we conducted two out-of-sample validations, assessing the degree to which the identified BMA prior-variance transformations served as a robust network-specific link between structural connectivity and prior variances. Utilizing both resting-state fMRI data—session-1 and -2 recordings—and structural connectivity from 50 healthy adults (24 female, age 22–35), out-of-sample validation mirrored the procedures utilized in the assessment of test-retest reliability. In this section, we present the out-of-sample validation results for session-1 data and refer the reader to the supporting information (SI Results, Figs. S4–S5) for the (similar) session-2 results.

**Fig. 4.**
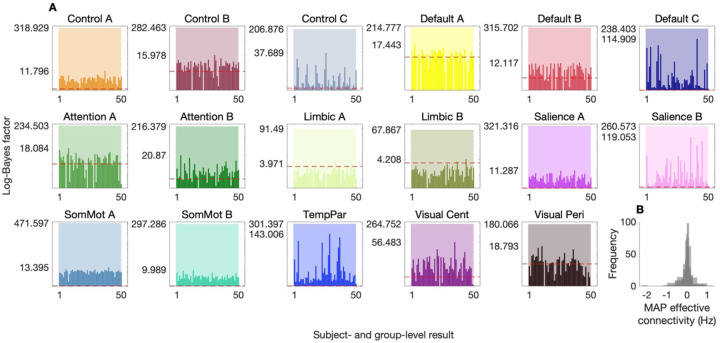
Out-of-sample validation of hierarchical empirical Bayes models. (**A**) Log-Bayes factors comparing hierarchical empirical Bayes models with structure-based priors (derived from out-of-sample BMA prior-variance transformations) to models with non-informative priors, across 17 effective connectivity brain networks. The semi-transparent bars represent the log-Bayes factor for group-level component of the model, while the opaque bars represent the log-Bayes factor for the subject-level component. The log-Bayes factors indicate substantially greater evidence for models incorporating structure-based priors. Note the use of a log-scaled y-axis, with a dashed red line indicating a log-Bayes factor of 3. Y-axis tick marks indicate the maximum increase in evidence at the subject level, and the increase in evidence at the group level. Log-scaling necessitated the exclusion of several decreases in evidence at the subject level (see Fig. S6 for alternative visualizations). Networks analyzed include control (Control A–C), default mode (Default A–C), attention (Attention A, B), limbic (Limbic A, B), salience (Salience A, B), somatomotor (SomMot A, B), temporoparietal (TempPar), and visual networks (Visual Cent, central; Visual Peri, peripheral). (**B**) Histogram of maximum a posteriori (MAP) group-level effective connectivity estimates for all networks.

Fig. 4A indicates substantially greater evidence for the hierarchical empirical Bayes models that utilized the out-of-sample BMA prior-variance transformations, relative to models with non-informative priors (note the use of a log-scaled y-axis). Note that for each network, we report the log-Bayes factor for both the group-level component of the model (semitransparent bars), in addition to log-Bayes factors for the subject-level component of the model (opaque bars). Note too that the smallest increase in evidence across the group-level component of hierarchical empirical Bayes models suggested that there is at least $e^{67}\approx 10^{29}$ times more evidence in favor of the model with structure-based priors, over the model with non-informative priors. Furthermore, for most networks, the introduction of these out-of-sample prior-variance transformations translated to an increase in evidence at the subject level and furnished MAP effective connectivity estimates within a plausible range (Fig. 4B).

### Criterion validity

**Fig. 5.**
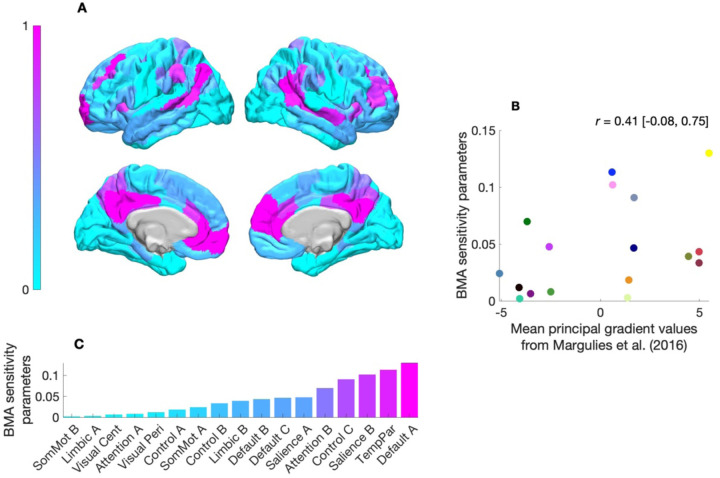
Coupling between structural and effective connectivity across functional brain networks. (**A**) Sensitivity parameters for each network-specific Bayesian model-average (BMA) variance transformation projected onto a pial surfaces using a gradient colormap. Higher sensitivity values (magenta) indicate greater coupling between structural connectivity and effective connectivity, with a peak in the posterior cingulate, temporal parietal and medial prefrontal cortices (corresponding to the Default A network). (**B**) Scatterplot showing the relationship between sensitivity parameters and mean principal gradient values from Margulies and colleagues’ study^12^. The relationship is quantified via the product-moment correlation $\left(r\right)$, with square-bracketed values indicating the 95% confidence interval. The trend suggests that the influence of structural connectivity on effective connectivity increases along an axis of decreasing functional specialization. (**C**) Bar plot of sensitivity parameters across networks, ranked by magnitude. Default mode (Default A) exhibited the highest sensitivity, while the somatomotor (SomMot B) network displayed the lowest sensitivity. Networks analyzed include control (Control A–C), default mode (Default A–C), attention (Attention A, B), limbic (Limbic A, B), salience (Salience A, B), somatomotor (SomMot A, B), temporoparietal (TempPar), and visual networks (Visual Cent, central; Visual Peri, peripheral).

To explore inter-network differences in the degree to which structural connectivity influenced—was linearly coupled with—effective connectivity, we examined the sensitivity parameters (the weights defining the gradient of the BMA prior-variance transformations in Fig. 3A). Here, we visualized these sensitivity parameters on a pial surface, with each parameter mapped to a gradient colormap (Fig. 5A). Fig. 4C indicates that structural connectivity had the greatest influence on effective connectivity in the default mode (Default A) network that comprised hub regions: the posterior cingulate and medial prefrontal cortices. Fig. 4C also shows that these sensitivity parameters were situated along an axis that describes an approximate unimodal (sensory) to transmodal (integrative) processing hierarchy^20^. In this way, notwithstanding that these networks have been examined separately, the results recapitulate a key aspect of a previously identified functional hierarchy which, in the human cortex, peaks in regions corresponding to the default mode network and reaches its nadir in somatomotor regions^12,21–23^. Fig. 4B shows a moderate positive correlation (r=0.41, non-significant) between sensitivity parameters and mean values from the principal gradient of functional connectivity described by Margulies and colleagues^12^. This trend suggests that structural connectivity’s influence on effective connectivity may increase as functional specialization decreases.

## Discussion

This study explored whether integrating structural connectivity into a hierarchical empirical Bayes model improved mapping of resting-state effective connectivity in terms of free energy (or approximate log-model evidence). Recently, Sokolov and colleagues introduced a method via which structural connectivity is integrated into a Bayesian RFX model of group-level effective connectivity^8,9^. Here, we built on this prior work in several ways. First, we incorporated Bayesian model averaging into our procedure to account for uncertainty in the selection of prior-variance transformations (rather than focusing on a single best transformation). Second, we examined the impact of utilizing structurally informed group-level effective connectivity as empirical priors for re-evaluating subject-level effective connectivity. Third, we establish the face validity of the hierarchical empirical Bayes model *in silico* and demonstrate that it is more accurate than a structurally informed MVAR model. Fourth, we demonstrated our procedure’s test-retest reliability and out-of-sample validity across resting-state networks. Finally, we showed that inter-network differences in the coupling between structural and effective connectivity recapitulate a well-known cortical hierarchy.

Our findings indicate that structural connectivity constrains resting-state effective connectivity, with the operative a priori assumption being that the probability of an interaction between two neuronal populations increases with the extent to which two regions are connected via nerve tracts detectable at the macroscale of MRI. However, this relationship between structural and effective connectivity does not appear to be uniform across the brain but rather appears to be modulated, hierarchically, along an approximate unimodal-transmodal axis^20^. The recapitulation of this well-known cortical hierarchy not only attests to the predictive validity of the hierarchical empirical Bayes model, but also hints at a deeper principle of brain organization. Namely, that the principal, unimodal-transmodal gradient of functional connectivity may be explained in terms of the influence that structural connectivity exerts on effective connectivity: an influence that appears to increase with decreasing functional specialization (in other words, as regions become more involved in integrating information across the brain).

Our results are aligned with those from studies that have integrated structural connectivity into effective and directed functional connectivity models via priors. Namely, previous work has shown that introducing a positive, monotonic mapping between structural connectivity and prior variances in the context of dynamic causal modeling and MVAR models, increases (approximate) model evidence^9,24–26^. More specifically, our work is aligned with prior work examining the impact of structure-based priors in group-level models^9,27^. It differs from this prior work, however, as previous investigations have not, per se, examined the impact of leveraging a structurally informed group-level effective connectivity to constrain subject-level effective connectivity, and have not validated structure-based priors with new, unseen data. Furthermore, rather than utilize a sigmoidal prior-variance transformation (per earlier studies^9,24^), we utilized a simpler and more interpretable linear function amenable to analytic Bayesian model averaging (unlike the sigmoid, linear functions are closed under linear combination, meaning the weighted average of linear functions remains linear).

The results of this study are also aligned with those that have involved embedding structural connectivity into MVAR-type models. Recently, Tanner and colleagues demonstrated that structurally informed directed functional connectivity obtained under such a model exhibited a hierarchical community structure^10^, and related models have demonstrated predictive validity across several neuropsychiatric conditions^28–30^. That said, such models preclude multi-hop directed functional connectivity (directed influences between region i to region j that are mediated via one or more relay regions). Our *in silico* analyses (Fig. 2E–F; Fig. S1B–C) suggest that this limitation may hinder the ability of these models to accurately characterize directionality (leading to higher sign errors, compared to the hierarchical empirical Bayes approach).

Our study has important implications. First, it underscores the importance of considering structural connectivity in effective connectivity, with findings suggesting that integrating structure-based priors into a hierarchical model of effective connectivity facilitates robust inference. Second, it offers a framework via which one can investigate the coupling between structural and effective connectivity, and how such coupling might be modified. The timeliness of such questions is thrown into sharp contrast considering recent evidence for disorder- and intervention-specific alterations in the hierarchical organization of brain function—with increased coupling (similarity) between functional and structural connectivity in various neuropsychiatric disorders (such as multiple sclerosis^31,32^), and psychedelics inducing an uncoupling of functional and structural connectivity^33,34^. Finally, the study demonstrates that the hierarchical empirical Bayes model outperforms a prominent alternative approach to characterizing directed influences between brain regions and has yielded findings that hint a mechanism via which the principal, unimodal-transmodal gradient of functional connectivity may emerge. Whether this gradient emerges from the differing constraint that the nerve tracts exert on interregional brain communication is an idea that warrants the attention of future investigations.

This study’s implications need to be considered in view of certain limitations. First, the key limitation to integrating structural connectivity into effective connectivity via structure-based priors is that the integration is somewhat heuristic, representing a statistical assumption rather than a process that maps to a biological mechanism^3^. That said, there are myriad ways in which such mechanistic information might be embedded into this hierarchical empirical Bayes model or incorporated into structural connectivity itself (for example, by adding biological annotations^35^). Second, the computational challenges inherent in inverting large DCMs necessitated that we invert DCMs for 17 resting-state effective connectivity networks separately, limiting our ability to investigate whole-brain coupling between structural and effective connectivity. Finally, owing to the inability of dwMRI to determine axonal directionality^36^, our procedure used symmetric structural connectivity, and thus for two given regions, equal priors were assigned for efferent and afferent effective connections. Future research can hope to address these issues by adapting methods that enable inference of whole-brain effective connectivity (such as regression dynamic causal modeling^37^) and exploring graph-theory-based methods of inferring asymmetric signaling from structural connectivity^38,39^.

In conclusion, our study highlights the critical role of structural connectivity in shaping effective connectivity, presenting—to our knowledge—the first evidence of network-dependent modulation of this relationship in humans. Using a novel hierarchical empirical Bayes method, we demonstrate that a positive, monotonic relationship between structural connectivity and the prior probability of non-zero effective connectivity generalizes across sessions and samples. The model’s criterion validity is supported by its recapitulation of a well-known cortical hierarchy, while its construct validity is evidenced by superior recovery of subject- and group-level effective connectivity compared to an alternative model. Taken together, these findings recommend a shift towards more integrative approaches in which the fusion of structural and effective connectivity could offer novel insights into functional integration in health and disease.

## Methods

### Data

Data used in this study were sourced from the HCP^19^. For acquisition and pre-processing details, in addition to details concerning tractography and the derivation of (parcellated) structural connectivity, we refer the reader to the supporting information (SI Methods). Furthermore, in the supporting information (SI Methods), we detail of the procedures used to derive simulation results (Fig. 2).

### Model

The hierarchical empirical Bayes model is used to characterize effective connectivity networks across and within subjects $\left(s=1,\ldots ,S\right)$, comprising i=1,…,n neuronal populations. At the subject level, the model builds on the well-known and well-validated DCM for resting-state fMRI (also known as spectral DCM)^4,40,41^. In this model, brain dynamics are represented by a continuous-time state-space model that describes how directed interactions between different neuronal populations drive changes in ensemble neuronal activity that, in turn, gives rise to observed BOLD signals, y:

(5) $$\dot{\text{x}}\left(t\right)=\text{Ax}\left(t\right)+\text{v}\left(t\right)\;\;\;\;\;\;\;\;\;\;\;\;\;\;\;\left(state\;equation\right)\;\text{y}\left(t\right)=h\left(\text{x}\left(t\right),{\text{θ}}_{h}\right)+\text{e}\left(t\right)\;\;\;\;\;\;\;\;\;\;\;\left(observation\;equation\right)$$

Here, $\text{x}\left(t\right)={\left[x_{1}\left(t\right),\ldots ,x_{n}\left(t\right)\right]}^{\text{T}}$ represents the unobserved (or latent) state of neuronal populations at time t, and h is the hemodynamic response function (based on the well-known balloon model^42,43^) with parameters ${\text{θ}}_{h}$. The transition matrix $\text{A}\in {\mathbb{R}}^{n\times n}$ encodes the intra- and inter-regional modulation of the rates of change in ensemble neuronal activity (quantifying the effective connectivity), in its diagonal and off-diagonal elements, respectively, with the former representing the intrinsic excitability of each region. In spectral DCM, both $\text{v}\left(t\right)$ and $\text{e}\left(t\right)$ are parameterized as power-law noise (see SI Methods for further details). Using the Fourier transform, 𝓕, the state-space model is projected into the spectral (frequency) domain, generating predictions about the cross-spectral density of the signal $\text{y}\left(t\right)$ at (rotational) frequency $\omega :{\text{G}}_{y}\left(\omega \right)=\left[𝓕\left\{\text{y}\left(t\right)\right\}𝓕{\left\{\text{y}\left(t\right)\right\}}^{†}\right]$. Here, † represents the conjugate transpose. The spectral equivalent of Equation 5 reads:

(6) $${\text{G}}_{y}\left(\omega \right)=\text{H}\left(\omega \right){\left(i\omega \text{I}-\text{A}\right)}^{-1}{\text{G}}_{v}\left(\omega \right){\left(-i\omega \text{I}-{\text{A}}^{T}\right)}^{-1}\text{H}{\left(\omega \right)}^{†}+{\text{G}}_{e}\left(\omega \right),$$

where $\text{H}\left(\omega \right)$ is the Fourier transform of the hemodynamic response function, I is an n-dimensional identify matrix, and ${\text{G}}_{v}\left(\omega \right)$ and ${\text{G}}_{e}\left(\omega \right)$ are the cross-spectral density of $\text{v}\left(t\right)$ and $\text{e}\left(t\right)$, at frequency ω, respectively. Importantly, the latent neuronal state in the frequency domain, $\left(\omega \right)$, has been factored out of the equations via the substitution $\text{X}\left(\omega \right)\text{X}{\left(\omega \right)}^{†}={\left(i\omega \text{I}-\text{A}\right)}^{-1}{\text{G}}_{v}\left(\omega \right){\left(-i\omega \text{I}-{\text{A}}^{T}\right)}^{-1}$. For a didactic introduction to these derivations, we refer the reader to work from Novelli and colleagues^44^. In the text that follows, we refer to Equation 6 as the first-, subject-level model $\Gamma^{\left(1\right)}\left({\text{A}}_{s}^{\left(1\right)}\right)={\text{G}}_{y,s}\left(\omega \right)$, where the subscript s denotes the subject-specificity of parameters and data, and the superscript (1) distinguishes these elements from higher levels of the hierarchy.

With the specification of a (multivariate) Gaussian prior over model parameters ${\text{θ}}_{s}$ (SI Methods), the statistical model for a given DCM $\left(\Gamma^{\left(1\right)}\right)$ is defined in terms of the joint probability distribution, $p\left({\text{G}}_{y,s}\left(\omega \right),{\text{θ}}_{s}\text{|}\Gamma^{\left(1\right)}\right)=p\left({\text{G}}_{y,s}\left(\omega \right)|{\text{θ}}_{s},\Gamma^{\left(1\right)}\right)\cdot p\left({\text{θ}}_{s}\text{|}\Gamma^{\left(1\right)}\right)$. This provides the necessary ingredients for model inversion via VBL (see Equation 2), under the assumption that the likelihood $p\left({\text{G}}_{y,s}\left(\omega \right)|{\text{θ}}_{s}\text{,}\Gamma^{\left(1\right)}\right)$ is Gaussian, reflecting independent and identically distributed (IID) additive Gaussian noise in the data.

With DCMs inverted for a given network, the (approximate) posteriors of interest—for effective connectivity—are moved into the hierarchical empirical Bayes model (Fig. 1)^7^. Stacking the vectorized posterior expectations for S subjects, ${\text{A}}^{\left(1\right)}=\left[\text{vec}\left({\text{A}}_{1}^{\left(1\right)}\right);\ldots ;\text{vec}\left({\text{A}}_{S}^{\left(1\right)}\right)\right]$, permits the following formulation:

(7) $$\begin{array}{l} {\text{A}}^{\left(2\right)}={\text{ε}}^{\left(3\right)}\;\;\;\;\;\;\;\;\;\;\;\;\;\;\;\;\;\left(third-level\;model\right)\\ {\text{A}}^{\left(1\right)}=\left(\text{X}⊗{\text{I}}_{n}\right){\text{A}}^{\left(2\right)}+{\text{ε}}^{\left(2\right)}\;\;\;\;\;\;\;\left(second-level\;model\right)\\ {\text{G}}_{y,s}\left(\omega \right)\text{=}\Gamma^{\left(1\right)}\left({\text{A}}_{s}^{\left(1\right)}\right)+{\text{ε}}_{s}^{\left(1\right)}\;\;\;\;\;\;\;\left(first-level\;model\right), \end{array}$$

where, at the third level, ${\text{A}}^{\left(2\right)}\in {\mathbb{R}}^{nn}$ is the group-level effective connectivity, modeled as error around a zero-mean expectation, represented by ${\text{ε}}^{\left(3\right)}$. At the second level, ${\text{A}}^{\left(1\right)}\in {\mathbb{R}}^{Sn^{2}}$ represents subject-level effective connectivity modeled via the design matrix $\text{X}=1_{S}$ —which here, in the absence of covariates, models the group mean—and ${\text{ε}}^{\left(2\right)}$ accounts for random deviations from ${\text{A}}^{\left(2\right)}$. The Kronecker product $\text{X}⊗{\text{I}}_{n}$ ensures that these RFX are applied separately to each connection in ${\text{A}}^{\left(1\right)}$. Finally, at the first level, ${\text{G}}_{y,s}\left(\omega \right)$ is the cross-spectral density of BOLD time-series data for subject s, given the subject-specific effective connectivity ${\text{A}}_{s}^{\left(1\right)}$, inferred under a given DCM ${\text{Γ}}^{\left(1\right)}$, with ${\text{ε}}_{s}^{\left(1\right)}$ encoding uncertainty around ${\text{A}}_{s}^{\left(1\right)}$.

Here, the variability at each level of the hierarchy— ${\text{ε}}_{s}^{\left(1\right)}∼𝓝\left(0,\sum_{s}^{\left(1\right)}\right)$, ${\text{ε}}^{\left(2\right)}∼𝓝\left(0,\sum^{\left(2\right)}\right)$ and ${\text{ε}}^{\left(3\right)}∼𝓝\left(0,\sum^{\left(3\right)}\right)$ —is zero-mean Gaussian, with $\sum_{s}^{\left(1\right)}$ encoding the posterior covariance of ${\text{A}}_{s}^{\left(1\right)}$, and $\sum^{\left(2\right)}$ parametrized in terms of its precision (SI Methods). At the third level, structural connectivity is incorporated into $\sum^{\left(3\right)}$ via the following:

(8) $${\text{φ}}^{\prime }\text{=}\frac{\left|\text{φ}\circ \left(1_{n}-\text{I}\right)\right|}{\max\left(\left|\text{φ}\circ \left(1_{n}-\text{I}\right)\right|\right)}\;\;\;\;\;\;\;\;\;\;\;\;\left(normalized\;structural\;connectivity\right)\;\sigma_{i,j}^{2}=\left\{\begin{array}{rr} \beta {\text{φ}}_{i,j}^{\prime }+\alpha , & i\neq j\\ \delta , & i=j \end{array}\;\;\;\;\;\;\;\;\;\;\;\right.\left(third-level\;variance\right)\;\sum^{\left(3\right)}=\text{diag}\left(\sigma_{i,j}^{2}\forall i,j\right)\;\;\;\;\;\;\;\;\;\;\;\;\;\;\;\;\;\left(third-level\;covariance\right)$$

Here, $\text{φ}\in {\mathbb{R}}_{\geq 0}^{n\times n}$ encodes the structural connectivity for an n-region network, $1_{n}-\text{I}$ is the complement of the n-dimensional identify matrix, and the normalized structural connectivity weights ${\text{φ}}_{i,j}^{\prime }$ from the i-th row and j-th column are transformed to produce variance terms $\sigma_{i,j}^{2}$ for corresponding group-level effective connections ${\text{A}}^{\left(2\right)}$, where i≠j. The parameters α and β determine the baseline variance (intercept) and sensitivity to structural connectivity (weight) of the transformation, respectively, and intraregional connections—where i=j —are set to δ. In the context of our analyses, non-informed models represent the case where β=0, α=1/2 and δ=1/64.

Assuming the data for each subject are conditionally independent, the full hierarchical empirical Bayes model, m, can be represented in terms of probability densities^45^:

(9) $$p\left({\text{G}}_{Y}\left(\omega \right),{\text{A}}^{\left(1\right)},{\text{A}}^{\left(2\right)}|m\right)=\Pi_{s=1}^{S}p\left({\text{G}}_{y,s}\left(\omega \right)|{\text{A}}_{s}^{\left(1\right)},m\right)\cdot p\left({\text{A}}^{\left(1\right)}|{\text{A}}^{\left(2\right)},m\right)\cdot p\left({\text{A}}^{\left(2\right)}|{\text{φ}}^{\prime }\text{,}m\right),$$

where ${\text{G}}_{Y}\left(\omega \right)=\left\{{\text{G}}_{y,1}\left(\omega \right),\ldots ,{\text{G}}_{y,S}\left(\omega \right)\right\}$ represents the cross-spectral densities for S subjects, and the first-level likelihood term represents the factorization of DCM posteriors (Fig. 1A). The second-level term $p\left({\text{A}}^{\left(1\right)}|{\text{A}}^{\left(2\right)},m\right)$ represents the (empirical) prior distribution over subject-specific parameters ${\text{A}}^{\left(1\right)}$ (the group-level posterior in Fig. 1B). and the third-level term $p\left({\text{A}}^{\left(2\right)}|{\text{φ}}^{\prime }\text{,}m\right)$ specifies the prior distribution of the group-level parameters ${\text{A}}^{\left(2\right)}$ (Fig. 1C).

Inverting the statistical model presented in Equation 9 proceeds via a two-step process. First, VBL is utilized to invert the group-level model and obtain the (approximate) posterior $q\left({\text{A}}^{\left(2\right)}|{\text{A}}^{\left(1\right)},m\right)$ (first insert, Fig. 1B). Subsequently, this posterior is treated as the prior for a reduced model, and subject-level models are re-evaluated using BMR (see Equation 4) to furnish (updated) posteriors $q\left({\text{A}}_{s}^{\left(1\right)}|{\text{A}}^{\left(2\right)},m\right)$ (second insert, Fig. 1B). Crucially, this two-step process yields estimates of both group- and subject-level free energy (permitting comparison of alternative group- and subject-level models).

### Procedures

In our study, for each of the 150 subjects, across 17 networks and two sessions, a spectral DCM was specified and inverted using VBL as implemented in the statistical parametric mapping toolbox (SPM12)^46^. We then inverted the hierarchical empirical Bayes model (Steps 1 and 2; Fig. 1) with non-informative third-level priors— β=0, α=1/2 and δ=1/64 —for each network within each dataset: the (session-1) test and (session-2) retest datasets $\left(N=100\right)$, and two (session-1 and -2) validation datasets $\left(N=50\right)$.

In the empirical face-validation phase, we used the test dataset DCMs and BMR to evaluate the (relative) evidence associated with different reduced (structurally informed) group-level models, varying the parametrization of the prior-variance transformation (Equation 8). In these analyses, α was sampled at 30 equidistant points across [−1/2, 1/2]. For each α, β was sampled at 30 equidistant points across [0, 1/2 − α]. Valid parameter combinations, satisfying α≥0 and $\alpha +\beta \geq 10^{-5}$, were retained, yielding a triangular sampling space (7,273 parameter regimes) with greater density near $\alpha +\beta =10^{-5}$. We then obtained the parameters of the BMA prior-variance transformation for each brain network following:

(10) $$f_{\text{BMA}}\left(x\right)=\sum_{k=1}^{K}\frac{{\text{BF}}_{k}}{\sum_{j=1}^{K}{\text{BF}}_{j}}\cdot \left(\beta_{k}x+\alpha_{k}\right),$$

where K is the is the total number of parameter combinations tested ($\alpha_{k}$, $\beta_{k}$ ), and the fraction represents the normalized Bayes factor for the k-th model comparison ${\text{BF}}_{k}=e^{\left(F_{mR,k}-F_{mF}\right)}$, where the k-th reduced (structurally informed) model is compared to the full (non-informed) model.

The network-specific BMA prior-variance transformations were applied out of session (in the retest dataset) and out of sample (in the validation datasets). This out-of-session and out-of-sample testing proceeded in the following fashion. First, having inverted a hierarchical empirical Bayes model—per network—under non-informative priors, the network-specific BMA prior-variance transformations were used to derive structure-based priors that were applied—using BMR—to re-evaluate the non-informed group-level models, and yield a reduced—structurally informed—posterior and reduced free energy per network. The reduced free energy for these structurally informed group-level models could then be compared to the free energy for the full (non-informed) group-level models (semitransparent bars, Fig. 4), and the reduced group-level posteriors could then serve as empirical priors that were applied—using BMR—to re-evaluate the non-informed subject-level models. In this way, we also obtained reduced—structurally informed—posteriors and reduced free energies at the subject level, permitting a comparison of the free energy for reduced (structurally informed) subject-level models to the free energy for subject-level models that were re-evaluated under the non-informed group-level model (opaque bars, Fig. 4).

### Data and materials availability

All data and software are publicly available. Our implementation of the hierarchical empirical Bayes model, and the *in silico* analyses and associated visualizations, are available at: https://github.com/mdgreaves/hierarchical-empirical-Bayes

## Supplementary Material

Supplement 1
