# Supplementary material for: Structurally informed resting-state effective connectivity recapitulates cortical hierarchy
Source: bioRxiv. 2025 Feb 28:2024.04.03.587831. Originally published 2024 Apr 4. Preprint. [Version 2] doi: 10.1101/2024.04.03.587831 (PMC11014588; doi:10.1101/2024.04.03.587831)
Supplement: Supplementary file 1 [file media-1.pdf]

Supporting information:
Structurally informed resting-state effective connectivity recapitulates cortical hierarchy

Authors
Matthew D. Greaves[1,2*], Leonardo Novelli[1,2], and Adeel Razi[1,2,3,4]

Affiliations
[1]School of Psychological Sciences, Monash University, Clayton, Victoria, Australia
[2]Monash Biomedical Imaging, Monash University, Clayton, Victoria, Australia
[3]Wellcome Centre for Human Neuroimaging, University College London, London, UK
[4]CIFAR Azrieli Global Scholars Program, CIFAR, Toronto, Ontario, Canada
*matthew.greaves@monash.edu

Overview
This supporting information (SI) provides additional details and analyses to complement the findings presented in the main text. It begins with an SI Methods section that explains the derivation of (variational) free energy and use Bayesian model reduction (BMR) in the context of Gaussian densities, and then elaborates on aspects of the hierarchical empirical Bayes model not covered in detail in the main text (including the parameterization of endogenous fluctuations, observation error, and the hemodynamic response). This is followed by a description of the *in silico* analyses, detailing data simulation, the definition of the structurally informed multivariate autoregressive (MVAR) model, and metrics used to evaluate model performance. The SI Methods section also provides an overview of the Human Connectome Project (HCP) data, including acquisition protocols, pre-processing pipelines, parcellation methods, and the construction of consensus structural connectivity.

The SI Results section includes additional visualizations, presenting additional results from *in silico* analyses, and results for the test-retest reliability and out-of-sample validation analyses in formats that complement those in the main text. To distinguish them from those presented in the main text, all equations, tables, and figures in the SI are labeled with an "S" prefix. Where terms or concepts are not defined within the SI itself, readers are referred to the relevant sections of the main text for further detail.

We note again that our implementation of the hierarchical empirical Bayes model, and the *in silico* analyses and associated visualizations, are available at: https://github.com/mdgreaves/hierarchical-empirical-Bayes.

SI Methods
Free energy and Bayesian model reduction
Under a mean field assumption, the (variational) free energy (main text, Equation 2), can be derived from the sufficient statistics—mean ($\boldsymbol{\mu}$) and covariance ($\boldsymbol{\Sigma}$)—of the prior, $p(\boldsymbol{\theta}|m) = \mathcal{N}(\boldsymbol{\mu}_\theta, \boldsymbol{\Sigma}_\theta)$, approximate posterior, $q(\boldsymbol{\theta}|m) = \mathcal{N}(\boldsymbol{\mu}_q, \boldsymbol{\Sigma}_q)$, and likelihood, $p(\boldsymbol{y}|\boldsymbol{\theta}, m) = \mathcal{N}(f(\boldsymbol{\theta}), \boldsymbol{\Sigma}_y)$, where $f(\boldsymbol{\theta})$ is the model's generated (synthetic) data and $\boldsymbol{\Sigma}_y$ is the estimated residual covariance. The relevant equation is as follows:

$$F_m = \frac{1}{2}\left[-\ln\left|\boldsymbol{\Sigma}_y\right| + \boldsymbol{\epsilon}_y^{\mathrm{T}}\boldsymbol{\Sigma}_y^{-1}\boldsymbol{\epsilon}_y - \ln\left|\boldsymbol{\Sigma}_\theta\right| + \boldsymbol{\epsilon}_\theta^{\mathrm{T}}\boldsymbol{\Sigma}_\theta^{-1}\boldsymbol{\epsilon}_\theta + \ln\left|\boldsymbol{\Sigma}_q\right| - \dim(\boldsymbol{\theta})\ln(2\pi)\right]. \quad \text{(S1)}$$

Here, $\ln|\cdot|$ is log-determinant function, $\boldsymbol{\epsilon}_y = \boldsymbol{y} - f(\boldsymbol{\theta})$, $\boldsymbol{\epsilon}_\theta = \boldsymbol{\mu}_q - \boldsymbol{\mu}_\theta$, and $\dim(\boldsymbol{\theta})$ is the dimensionality of $\boldsymbol{\theta}$. This formulation permits the optimization of $\boldsymbol{\mu}_q$ and $\boldsymbol{\Sigma}_q$ such that free energy is maximized, and the approximate posterior distribution optimally balances model fit and model complexity[1].

BMR enables one to adjust the adjust the sufficient statistics of the prior distribution, $p(\boldsymbol{\theta}|m_R) = \mathcal{N}(\boldsymbol{\mu}_{\theta,R}, \boldsymbol{\Sigma}_{\theta,R})$, and obtain the free energy, $F_{m,R}$, and posterior distribution, $q(\boldsymbol{\theta}|m_R) = \mathcal{N}(\boldsymbol{\mu}_{q,R}, \boldsymbol{\Sigma}_{q,R})$, for a

reduced (alternative) model, m_R, from a full (parent) model, m, analytically (without the need for optimization). Specifically, BMR yields the reduced model as follows[2]:

$$
\begin{aligned}
F_{m,R} &= \frac{1}{2}\ln\left|\boldsymbol{\Sigma}_{\theta,R}^{-1}\boldsymbol{\Sigma}_q^{-1}\boldsymbol{\Sigma}_{q,R}\boldsymbol{\Sigma}_\theta\right| \\
&\qquad - \frac{1}{2}\big(\boldsymbol{\mu}_q^{\mathrm{T}}\boldsymbol{\Sigma}_q^{-1}\boldsymbol{\mu}_q + \boldsymbol{\mu}_{\theta,R}^{\mathrm{T}}\boldsymbol{\Sigma}_{\theta,R}^{-1}\boldsymbol{\mu}_{\theta,R} - \boldsymbol{\mu}_\theta^{\mathrm{T}}\boldsymbol{\Sigma}_\theta^{-1}\boldsymbol{\mu}_\theta \\
&\qquad - \boldsymbol{\mu}_{q,R}^{\mathrm{T}}\boldsymbol{\Sigma}_{q,R}^{-1}\boldsymbol{\mu}_{q,R}\big) + F_m \\
\boldsymbol{\mu}_{q,R} &= \boldsymbol{\Sigma}_{q,R}\big(\boldsymbol{\Sigma}_q^{-1}\boldsymbol{\mu}_q + \boldsymbol{\Sigma}_{\theta,R}^{-1}\boldsymbol{\mu}_{\theta,R} - \boldsymbol{\Sigma}_\theta^{-1}\boldsymbol{\mu}_\theta\big) \\
\boldsymbol{\Sigma}_{q,R} &= \big(\boldsymbol{\Sigma}_q^{-1} + \boldsymbol{\Sigma}_{\theta,R}^{-1} - \boldsymbol{\Sigma}_\theta^{-1}\big)^{-1}.
\end{aligned} \tag{S2}
$$

Here, per this study, the diagonal of $\boldsymbol{\Sigma}_{\theta,R}$ might encode the prior variance for effective connectivity parameters that depend on structural connectivity. By updating priors in this way, BMR allows structurally informed models to be efficiently evaluated *post hoc*.

Hierarchical empirical Bayes model

To fully specify the hierarchical empirical Bayes model, it is necessary to detail the form of endogenous fluctuations $\boldsymbol{v}(t)$, observation error $\boldsymbol{e}(t)$, the hemodynamic function h, and the second-level prior covariance $\boldsymbol{\Sigma}^{(2)}$. Regarding the state equation and observation equations used in the (first-level) dynamic causal model (DCM; main text, Equation 5), both $\boldsymbol{v}(t)$ and $\boldsymbol{e}(t)$ are parameterized power-law noise such that the power spectral density (PSD) of endogenous fluctuations and observation error for the i-th neuronal population (or region)—$\boldsymbol{G}_{v,ii}(\omega)$ and $\boldsymbol{G}_{e,ii}(\omega)$, respectively—are given by the following equations:

$$
\begin{aligned}
\boldsymbol{G}_{v,i,i}(\omega) &= \alpha_v \omega^{-\beta_v}, \\
\boldsymbol{G}_{e,i,i}(\omega) &= \big(\gamma_e \cdot \alpha_{e,i}\big) \cdot \frac{\omega^{-\beta_e/2}}{\sum_\omega \omega^{-\beta_e/2}}.
\end{aligned} \tag{S3}
$$

Here, the $\{\alpha_v, \alpha_{e,i}\}$ and $\{\beta_v, \beta_e\}$ parameters control the amplitude and power-law decay (or slope) of the PSD, respectively, with the amplitude of region-specific observation error normalized by γ_e. Note that—per the default used in the statistical parametric mapping (SPM) toolbox[3]—in the absence of region-specific neuronal fluctuations, $\boldsymbol{G}_{v,i,i}(\omega)$ is identical for all regions.

To illustrate the general form of the cross-spectral density (CSD) matrix, consider the CSD matrix for the blood-oxygen-level-dependent (BOLD) signal, $\boldsymbol{G}_y(\omega)$, which—in a model with n neuronal populations—can be represented as:

$$
\boldsymbol{G}_y(\omega) = \begin{bmatrix} \boldsymbol{G}_{y,1,1}(\omega) & \cdots & \rho_{y,1,n}(\omega)\cdot\sqrt{\boldsymbol{G}_{y,n,n}(\omega)\boldsymbol{G}_{y,1,1}(\omega)} \\ \vdots & \ddots & \vdots \\ \rho_{y,n,1}(\omega)\cdot\sqrt{\boldsymbol{G}_{y,1,1}(\omega)\boldsymbol{G}_{y,nn}(\omega)} & \cdots & \boldsymbol{G}_{y,n,n}(\omega) \end{bmatrix}, \tag{S4}
$$

where the off-diagonal elements $\boldsymbol{G}_{y,i,j}(\omega)$ are the CSDs, which depend on the coherence (spectral correlation) $\rho_{y,i,j}(\omega)$ between PSDs $\boldsymbol{G}_{y,i,i}(\omega)$ and $\boldsymbol{G}_{y,j,j}(\omega)$. The CSD matrix for $\boldsymbol{G}_v(\omega)$ and $\boldsymbol{G}_e(\omega)$ follows the same form; however, in spectral DCM, the off-diagonal elements of $\boldsymbol{G}_v(\omega)$ and $\boldsymbol{G}_e(\omega)$ are assumed to be zero.

Next, $h(\boldsymbol{x}(t), \boldsymbol{\theta}_h)$, in the observation equation, represents a system of equations—the well-known adaptation of the Balloon model[4]— that derives the (multivariate) BOLD signal $\boldsymbol{y}(t)$ from neuronal states $\boldsymbol{x}(t)$ as follows:

$$\begin{aligned}
\dot{\boldsymbol{s}}(t) &= \boldsymbol{x}(t) - k_h \cdot \boldsymbol{s}(t) - \gamma_h \cdot (\boldsymbol{f}(t) - 1), && \text{(vasodilatory signal)} \\
\dot{\boldsymbol{f}}(t) &= \boldsymbol{s}(t), && \text{(blood flow induction)} \\
\dot{\boldsymbol{b}}(t) &= \frac{1}{\boldsymbol{\tau}} \cdot \boldsymbol{f}(t) - \boldsymbol{b}(t)^{1/\alpha_h}, && \text{(blood volume)} \\
\dot{\boldsymbol{q}}(t) &= \frac{1}{\boldsymbol{\tau}} \cdot \left(\frac{\boldsymbol{f}(t) \cdot \left(1 - (1 - E_0)^{1/\boldsymbol{f}(t)}\right)}{E_0} - \boldsymbol{q}(t) \cdot \boldsymbol{q}(t)^{1/\alpha_h - 1} \right), && \text{(deoxyhemoglobin content)} \\
\boldsymbol{y}(t) &= V_0 \cdot \left[k_1 (1 - \boldsymbol{q}(t)) + k_2 \left(1 - \frac{\boldsymbol{q}(t)}{\boldsymbol{b}(t)}\right) + k_3 (1 - \boldsymbol{b}(t)) \right]. && \text{(BOLD signal)}
\end{aligned} \tag{S5}$$

In the implementation utilized in this study, $\boldsymbol{\theta}_h = \{k_h, \boldsymbol{\tau}, \epsilon\}$ are free parameters. Reading from the first line down: k_h is the decay rate of the vasodilatory signal, $\gamma_h = 0.32$ regulates feedback of blood flow, $\boldsymbol{\tau} = [\tau_1, \dots, \tau_n]$, are region-specific transit times, $\alpha_h = 0.32$ is Grubb's (vessel stiffness) exponent, and $E_0 = 0.4$ and $V_0 = 4$ represent the resting oxygen extraction fraction and the resting venous blood volume fraction, respectively. In the last line, coefficients k_1, k_2, and k_3 represent the contributions of the intravascular and extravascular compartments to the BOLD signal:

$$\begin{aligned}
k_1 &= 4.3 \cdot v_0 \cdot E_0 \cdot TE, \\
k_2 &= \epsilon \cdot r_0 \cdot E_0 \cdot TE, \\
k_3 &= 1 - \epsilon,
\end{aligned} \tag{S6}$$

where $v_0 = 40.3$ is the frequency offset at the outer surface of magnetized vessels, $TE = 0.04$ is the echo time, $r_0 = 25$ is the slope of the intravascular relaxation rate, and ϵ is the ratio of intra- to extra-vascular signal contributions.

Finally, moving to the second level of the hierarchical model: the random effects (RFX), $\boldsymbol{\varepsilon}^{(2)} \sim \mathcal{N}(0, \boldsymbol{\Sigma}^{(2)})$, are parameterized in terms of a scaled precision component:

$$\boldsymbol{\Sigma}^{(2)^{-1}} = \boldsymbol{I}_S \otimes (\boldsymbol{Q}_0 + e^{-\gamma} \boldsymbol{Q}_1) \tag{S7}$$

where $\boldsymbol{Q}_0 = \boldsymbol{\Sigma}_{non}^{(3)^{-1}} e^{-8}$ is the lower-bound precision derived from the non-informed, third-level prior covariance matrix, $\boldsymbol{\Sigma}_{non}^{(3)}$ (obtained under parameters $\beta = 0$, $\alpha = 1/2$ and $\delta = 1/64$; main text, Equation 8), $\boldsymbol{I}_S$ is the S-dimensional identity matrix, and $\boldsymbol{Q}_1 = \boldsymbol{\Sigma}_{non}^{(3)^{-1}} 16$, per the default used in the SPM toolbox[3]. Here, γ is a free parameter inferred from the data. In Table S1, we report the prior expectation (mean) and uncertainty (variance) for all free parameters.

Parameter	Description	Prior mean	Prior variance
$\ln\left(-2\cdot \boldsymbol{A}^{(1)}_{s,i,i}\right)$	First-level intra-regional effective connectivity	$-1/2$	δ
$\boldsymbol{A}^{(1)}_{s,i,j}$	First-level inter-regional effective connectivity	$1/128$	$1/2$
$\ln\alpha_v$	Amplitude of endogenous fluctuations	0	$1/64$
$\ln\beta_v$	Slope of endogenous fluctuations	0	$1/64$
$\ln\alpha_{e,i}$	Amplitude of observation error	0	$1/64$
$\ln\beta_e$	Slope observation error	0	$1/64$
$\ln\gamma_e$	Global amplitude normalization	0	$1/64$
$\ln k_h$	Decay rate of the vasodilatory signal	0	$1/256$
$\ln\tau_i$	Transit time	0	$1/256$
$\ln\epsilon$	Ratio of intra- to extra-vascular signal	0	$1/256$
$\ln\left(-2\cdot \boldsymbol{A}^{(2)}_{i,i}\right)$	Second-level intra-regional effective connectivity	$-1/2$	δ
$\boldsymbol{A}^{(2)}_{i,j}$	Second-level inter-regional effective connectivity	$1/128$	$f(\alpha,\beta,\boldsymbol{\varphi}')$
γ	Scaling factor for precision of random effects	0	$1/16$

Table S1. Prior mean and variance for free parameters utilized in the hierarchical empirical Bayes model. Notation is consistent with that utilized in the main text. Note that the priors for group- and subject-level effective connectivity—$\boldsymbol{A}^{(2)}$ and $\boldsymbol{A}^{(1)}_s$, respectively—do not differ from one another if the group-level model is non-informed. If the model is structurally informed, the second-level inter-regional effective connectivity is a function of normalized structural connectivity, and the parameters governing the prior-variance transformation. The derivation of structurally informed priors is detailed at length elsewhere (main text, Equation 8).

Note that for simplicity, we report (in the main text) that priors for inter-regional effective connectivity have a zero mean. This reporting convention is consistent with earlier related studies[5–7]. In practice, the prior mean reflects a weak positive bias toward (non-zero) effective connectivity[8], a choice that ensures numerical stability during model inversion. Note too that here, $\delta = 1/64$ for all analyses.

In silico analyses

To evaluate the face validity of the hierarchical empirical Bayes model, we conducted a parameter recovery analysis. In addition, we compared the model with a structurally informed MVAR model. These analyses involved the simulation of group- and subject-level effective connectivity, neuronal dynamics, and BOLD signals.

First, we obtained a random, sparse, symmetric matrix $\boldsymbol{\varphi} \in \mathbb{R}^{n\times n}_{\geq 0}$ with density d. Here, $\boldsymbol{\varphi}$ satisfied the condition that at least one region is completely unconnected: there exists at least one row (column) that is entirely zero. We obtained the normalized structural connectivity according to the following:

$$\boldsymbol{\varphi}' = \frac{|\boldsymbol{\varphi} \circ (\mathbf{1}_n - \boldsymbol{I})|}{\max(|\boldsymbol{\varphi} \circ (\mathbf{1}_n - \boldsymbol{I})|)}, \tag{S8}$$

where, $\mathbf{1}_n - \boldsymbol{I}$ is the complement of the n-dimensional identify matrix. We then obtained group- and subject-level effective connectivity—$\boldsymbol{A}^{(2)}$ and $\boldsymbol{A}^{(1)}_s$, respectively—via the following:

$$\begin{aligned}\boldsymbol{A}^{(2)} &= \ln\left(-2\cdot\left(-{}^{1}\!/_{2} + \sqrt{\delta}\cdot \boldsymbol{I}\circ \boldsymbol{N}\right)\right) + \sqrt{(\beta\varphi' + \alpha)}\cdot(\mathbf{1}_n - \boldsymbol{I})\circ \boldsymbol{N},\\ \boldsymbol{A}^{(1)}_s &= \boldsymbol{A}^{(2)} + \ln\left(-2\cdot\left(-{}^{1}\!/_{2} + \sqrt{\delta}\cdot \boldsymbol{I}\circ \boldsymbol{Z}_s\right)\right) + \sqrt{\delta}\cdot(\mathbf{1}_n - \boldsymbol{I})\circ \boldsymbol{Z}_s.\end{aligned} \tag{S9}$$

Here, $\boldsymbol{N} \in \mathbb{R}^{n\times n}$ represents a zero-mean Gaussian noise process (Ziggurat method[9]) where each element is independently drawn from $\mathcal{N}(0,1)$, and $\boldsymbol{Z}_s \in \mathbb{R}^{n\times n}$ represents an independent zero-mean Gaussian noise process for $s = 1, \dots, S$ instantiations (simulated subjects). The parameters α and β linearly transform the

normalized structural connectivity ($\boldsymbol{\varphi}'$) and scale the variance of the process (for off-diagonal regions), and δ scales the variance for the diagonal group-level connections, in addition to the diagonal and off-diagonal subject-level connections. Note the log-normal sampling for diagonal connections ensured that they conformed the biophysically motivated convention implemented in the SPM toolbox[3].

Next, for each instantiation, we modeled endogenous fluctuations as a first-order autoregressive process parametrized per Razi and colleagues' construct validation study[10]:

$$\boldsymbol{v}[k] = {}^{1}\!/_{2}\,\boldsymbol{v}[k-1] + \boldsymbol{u}_v[k], \qquad \boldsymbol{u}_v[k] \sim \mathcal{N}\left(0, {}^{1}\!/_{16}\right), \tag{S10}$$

Where $\boldsymbol{v}[k] \in \mathbb{R}^n$ is a vector representing the neuronal activity endogenous to neuronal populations, and $k = 1, \dots, K$ indexes the discrete time steps. Leveraging standard continuous-to-discrete transformations whereby continuous-time dynamics are sampled at discrete intervals defined by the repetition time (TR), the state and observation equations (main text, Equation 5) were used to generate discrete-time BOLD-like time-series data of length K.

For region i-th BOLD-like time series, we then obtained region-specific observation error $\boldsymbol{e}_i[k] \in \mathbb{R}$:

$$\boldsymbol{e}_i[k] = {}^{1}\!/_{2}\,\boldsymbol{e}_i[k-1] + \boldsymbol{u}_{e,i}[k], \qquad \boldsymbol{u}_{e,i}[k] \sim \mathcal{N}\left(0, \frac{\sigma_{y,i}^2}{\mathrm{SNR}}\right), \tag{S11}$$

where $\sigma_{y,i}^2$ is the variance of the (simulated) BOLD signal for region i, and SNR is the desired signal-to-noise ratio. Note that in the main text, we focused on results derived using $\mathrm{SNR} = 1$ (Fig. 1); however, in a macro F1-based analysis (see SI Results), this SNR was varied $\{1, 5, 10, 50, 100, 200\}$. In Table S2, we report all other hardcoded parameter values utilized.

Parameter	Description	Value(s)
n	Number of regions	6
S	Number of instantiations (simulated subjects)	50
d	Density of (random) structural connectivity	$2/3$
K	Number of observations (scans)	10^3
TR	Temporal resolution (repetition time)	1
α	Baseline variance of group-level, inter-regional effective connectivity	$1/16$
β	Sensitivity of group-level variance to structural connectivity	$1/10$
δ	Variance of subject- and group-level intra-regional effective connectivity	$1/64$
$\ln k$	Decay rate of the vasodilatory signal	0
$\ln \tau_i$	Transit time	$\sim \mathcal{N}(0, e^{-8})$
$\ln \epsilon$	Ratio of intra- to extra-vascular signal contributions	0

Table S2. Hardcoded parameter values used in simulations. Notation is consistent with that utilized in the main text. Note that we utilized one set of (random) transit times for all instantiations (simulated subjects). Simulations used the default random number generator in MATLAB to ensure reproducibility.

Finally, we inverted the hierarchical empirical Bayes model described in detail in the main text (and preceding section), in addition to estimating—using ordinary least squares—directed functional connectivity via the following structurally informed MVAR model:

$$\boldsymbol{y}[k] = (\boldsymbol{W} \circ \boldsymbol{\varphi}^*)\boldsymbol{y}[k-1] + \boldsymbol{c}, \qquad \boldsymbol{\varphi}_{ij}^* = [\varphi_{ij}' > 0], \tag{S12}$$

where $\boldsymbol{y}[k] \in \mathbb{R}^n$ is a vector representing the observed BOLD activity, $\boldsymbol{W} \in \mathbb{R}^{n \times n}$ is the weight matrix capturing the degree to which the past activity of one region predicts the future activity of another, and $\boldsymbol{\varphi}^* \in \{0,1\}^{n \times n}$ is a binarization of the normalized structural connectivity matrix which constrains the directed functional connectivity modeled by $\boldsymbol{W}$**.** The Iverson bracket notation $[\boldsymbol{\varphi}_{ij}' > 0]$ is used to indicate that all non-zero elements are set to 1. Finally, $\boldsymbol{c} \in \mathbb{R}^n$ is the offset (bias) vector, which accounts for baseline activity in each region.

Although this model is described as a structurally informed MVAR model as a useful shorthand, it departs from standard MVAR formulations in one important way. Namely, the model omits self-connections ($\varphi_{ii}^* = 0$), meaning that it does not include autoregressive terms for each region's own past activity. Despite this deviation, the term "MVAR" effectively conveys the model's underlying framework with regards to leveraging lagged dependencies between variables.

We present results that make use of the root mean squared error (RMSE) and a macro F1-score to evaluate model performance. The RMSE is given by:

$$\mathrm{RMSE} = \sqrt{\frac{1}{N}\sum_{i=1}^{N}\left(\boldsymbol{A} - \widehat{\boldsymbol{A}}\right)^2}, \tag{S13}$$

where $\boldsymbol{A}$ are the ground-truth values, $\widehat{\boldsymbol{A}}$ are the estimated values (taken from $\boldsymbol{W}$ in Equation S12, for example), and $N = n^2$ is the total number of connections in the effective connectivity network. The macro F1-socre was given by:

$$F_{\text{macro}} = \frac{1}{|\mathcal{C}|}\sum_{c\in\mathcal{C}} F_c\,,\ \ F_c = \frac{2\cdot\text{Precision}_c\cdot\text{Recall}_c}{\text{Precision}_c+\text{Recall}_c}, \tag{S14}$$

where $\mathcal{C}$ represents the set of classes (positive, negative and absent effective connections), and precision and recall for class c are:

$$\text{Precision}_c = \frac{\text{TP}_c}{\text{TP}_c+\text{FP}_c},\ \ \text{Recall}_c = \frac{\text{TP}_c}{\text{TP}_c+\text{FN}_c}, \tag{S15}$$

where TP_c, FP_c and FN_c are the true positives, false positives and false negatives for class c, respectively. To account for floating-point precision and small numerical discrepancies, a threshold of $\zeta = 10^{-5}$ was utilized in these calculations. This threshold ensured that values close to zero were appropriately classified into one of three classes: positive $(+1)$, negative (-1), or absent (0). Specifically, values were classified as positive if they exceed ζ, negative if they are less than $-\zeta$, and absent if they lied within $[-\zeta, \zeta]$.

Data

Data used in this study were sourced from the HCP[11]. Test (face-validation) and retest datasets included data from 100 healthy adults (54 female, age 22–35) acquired on two different days (session 1 and session 2, respectively)[*]; and two out-of-sample validation datasets included data from 50 healthy adults (24 female, age 22–35) acquired on two different days[†].

Data acquisition and pre-processing

Data comprised both diffusion-weighted MRI (dwMRI) and resting-state functional MRI (fMRI) scans from each participant. dwMRI data were obtained using a spin-echo echo-planar imaging (EPI) sequence constrained by a repetition time (TR) of 5,520 ms, echo time (TE) of 89.5 ms, a flip angle of 78 degrees, and a multiband factor of 3. The field of view (FOV) was set to 210 mm in the readout (RO) direction and 180 mm in the phase encoding (PE) direction, with a resolution matrix of 168 x 144 (RO x PE). Per subject, the data consisted of 111 slices with a 1.25 mm isotropic voxel size, and diffusion-weighted measurements were performed with b-values of 1,000, 2,000, and 3,000 s/mm2, and six b0 scans were also acquired for signal normalization.

The fMRI data were acquired using a gradient-echo EPI sequence constrained by a TR of 720 ms, a TE of 33.1 ms, and a flip angle of 52 degrees, and employed a multiband factor of 8. The FOV for rfMRI data was 208 x 180 mm (RO x PE), with a resolution matrix of 104 x 90 (RO x PE). Per participant, the data consisted of 72 slices with an isotropic voxel size of 2 mm. The entire resting-state fMRI scanning session lasted for 14 m 33 s, yielding 1,200 time points of functional brain activity-related data. The HCP protocol includes data acquired using two phase-encoding directions: left-to-right (L-R) and right-to-left (R-L). For our study, we used the L-R phase direction in each dataset.

The HCP minimally pre-processed pipeline (v3.19.0) was employed for pre-processing the data, with denoising of the functional timeseries data completed using the independent component analysis-based X-

[*]Subject identifiers: 100206, 100307, 100408, 100610, 101006, 101107, 101309, 101915, 102109, 102311, 102513, 102614, 102715, 102816, 103010, 103111, 103212, 103414, 103515, 103818, 104012, 104416, 104820, 105014, 105115, 105216, 105620, 105923, 106016, 106319, 106521, 106824, 107018, 107321, 107422, 107725, 108020, 108121, 108222, 108323, 108828, 109123, 109830, 111009, 111211, 111312, 111413, 111514, 111716, 112112, 112314, 112516, 112920, 113215, 113316, 113619, 113922, 114217, 114419, 114621, 114823, 115017, 115320, 115724, 115825, 116221, 116524, 116726, 117021, 117122, 117324, 117930, 118124, 118225, 118528, 118730, 118831, 118932, 119025, 119732, 119833, 120111, 120212, 120414, 120515, 120717, 121416, 122317, 122418, 122620, 122822, 123117, 123420, 123521, 123723, 123925, 124624, 124826, 125222, 125424.

[†]Subject identifiers: 108525, 110007, 110411, 110613, 119126, 121618, 121921, 124220, 124422, 125525, 126325, 129634, 129937, 130013, 130114, 130316, 130417, 130720, 131217, 131419, 131823, 131924, 132017, 133019, 133625, 133928, 134021, 134223, 134627, 134728, 134829, 135124, 135225, 135528, 135629, 135730, 135932, 136126, 136227, 136631, 137532, 137633, 138130, 138231, 138332, 139839, 140117, 140319, 140420, 140824.

noiseifier (FIX) algorithm[12]. We refer the reader to the extensive HPC documentation for further details regarding these acquisition and pre-processing steps[13].

Tractography

Deterministic tractography was performed utilizing the fiber assignment by continuous tractography (FACT) algorithm via MRtrix3[14,15]. First, at each voxel, a single fiber orientation was modelled by the primary eigenvector of a diffusion tensor fitted to DWI data using iterative least squares. Second, 10 million streamlines were propagated voxel-wise, following the direction of the most aligned (colinear) fiber orientations. Streamlines were dynamically seeded from regions in which streamlines were sparse (relative to the fiber density suggested by the underlying fiber orientation distributions)[16], and either terminated at the gray matter-white matter interface, or at a point where fractional anisotropy (FA) fell below 0.2, curvature exceeded 45 degrees, or length exceeded 250 mm. Finally, these streamlines were downsampled by a factor of 5, and anatomically constrained tractography (ACT) was used to improve their biological plausibility[17].

Network parcellation

In our study, fMRI and tractography data were parcellated into distinct regions of interest (ROIs) using the 200-region cortical atlas developed by Schaefer and colleagues[18]. This atlas, which was generated based on a gradient-weighted Markov random field model and resting-state functional connectivity, can be organized according to 17 resting-state networks identified by Yeo and colleagues[19]. Each network, on average, consists of approximately 12 distinct regions, which permitted us to effectively investigate network-level dynamics across the cortex while managing the computational complexity associated with inverting large network models.

Tractography data were parcellated using MRtrix3 and a radial search procedure in which streamlines were assigned to parcels within a 5-mm radius of their endpoints. The fMRI data were parcellated—averaged—using standard HCP workbench command tools. Parcellation yielded (200 x 200) structural connectivity matrices, and (200 x 1,200) fMRI data matrices (capturing time-varying activity across 200 brain regions over 1,200 time points).

With regards to network nomenclature, the 17 networks in Schaefer and colleagues' atlas include: control (Control A–C), default mode (Default A–C), attention (Attention A, B), limbic (Limbic A, B), salience (Salience A, B), somatomotor (SomMot A, B), temporoparietal (TempPar), and visual networks (Visual Cent, central; Visual Peri, peripheral). It is important to note that these network labels are heuristic in nature: they should be considered as providing a useful but simplified representation of the functional roles that these networks play[19].

Structural connectivity

Tractography exhibits well-documented biases whereby short-range connections are overrepresented, overshadowing longer-distance tracts; and larger brain regions appear disproportionately connected (connectivity is positively correlated with regional surface area)[20]. To account for these issues, for both the test-retest and validation samples, we generated distance-based consensus structural connectivity matrices per the approach described by Betzel and colleagues[21]. These matrices retained the distributions of within-hemisphere and between-hemisphere connection lengths (as approximated via the Euclidean distances between parcel centroids). Connections were divided into 32 distance bins, and edges were selected for inclusion in the group matrix based on their consistency across subjects within each bin. The resultant unweighted consensus matrix was multiplied, elementwise, with an average matrix (the mean connectivity values between all pairs of regions across subjects' data). The resultant weighted consensus matrix was normalized by parcel surface area, yielding a group-level connectome of normalized connection weights.

Cortical gradient of functional connectivity

Margulies and colleagues applied a diffusion embedding algorithm to fMRI data to extract latent components, referred to as gradients, underlying functional connectivity[22]. The principal gradient separated unimodal and transmodal regions along an approximate sensory-fugal axis[23], with somatomotor and default mode regions situated at opposite ends. Leveraging this principal gradient as a quantitative description of this spectrum, we downsampled the gradient from vertex to network resolution by averaging gradient values per each of the 17 Schaefer networks and compared these mean principal gradient values to the sensitivity parameters associated with network-specific BMA prior-variance transformations.

SI Results

Here, we show additional results for *in silico* analyses, that concern the subject level (Fig. S1A-B), and group level (Fig. S1C), prior to providing visualizations of the test-retest reliability results (Fig. S2-S3) and the session-2 out-of-sample validation results (Fig. S4-S5). In the main text, log scaling applied to Fig. 3A necessitated the exclusion of several decreases in evidence at the subject level. Thus, we provide an alternative visualization for this result (Fig. S6), in addition to providing an alternative visualization of the test-retest reliability and session-2 out-of-sample validation results.

Here, Fig. S1A shows the RMSE assessed across the simulated subjects (SNR of 1). Although both the MVAR and hierarchical empirical Bayes model tended to exhibit relatively low error, the latter model demonstrated a tendency toward increased error in certain instances. In contrast, the MVAR model yielded more stable RMSE values across subjects. Fig. S1B shows the polarity error (the percentage of sign errors) assessed across the simulated subjects (SNR of 1), with results indicating that the MVAR model consistently struggled to correctly capture the directionality of connections. Finally, when assessed using the macro F1-score across varying SNRs (Fig. S1C), the group-level estimates obtained under the hierarchical empirical Bayes model consistently outperformed those obtained under the MVAR model.

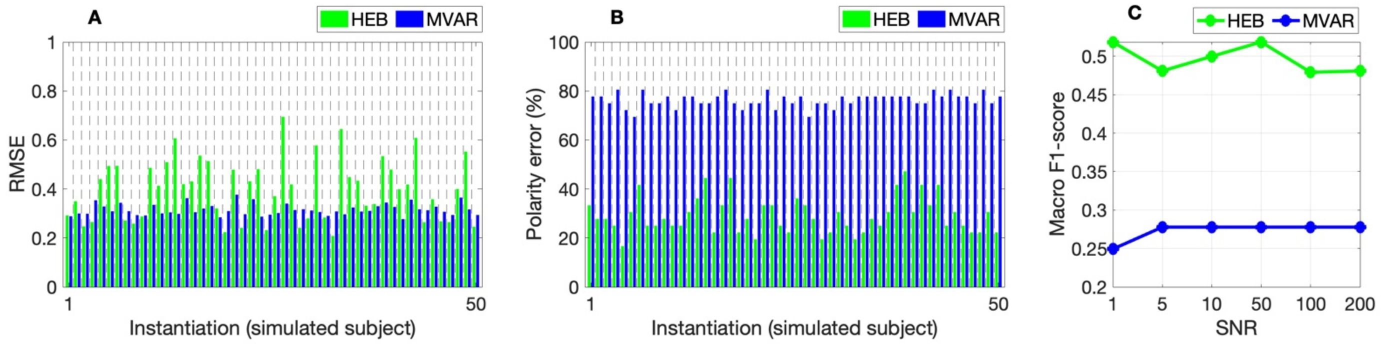

Fig. S1. Additional results for *in silico* evaluation of hierarchical empirical Bayes model. (**A**) Bar plot of root mean squared error (RMSE) comparing true and estimated subject-level effective connectivity characterized via the hierarchical empirical Bayes (HEB) model (green bars) and multivariate autoregressive (MVAR) model (blue bars) across 50 instantiations, highlighting the more stable RMSE values for the latter model. (**B**) Bar plot of polarity error (the percentage of sign errors) comparing the true and estimated subject-level effective connectivity characterized via the HEB model (green bars) and MVAR model (blue bars) across 50 instantiations, highlighting the greater performance of the former model. (**C**) Macro F1-scores demonstrate that the HEB model more accurately classifies positive, negative, and absent group-level effective connections across varying signal-to-noise ratio (SNR) levels.

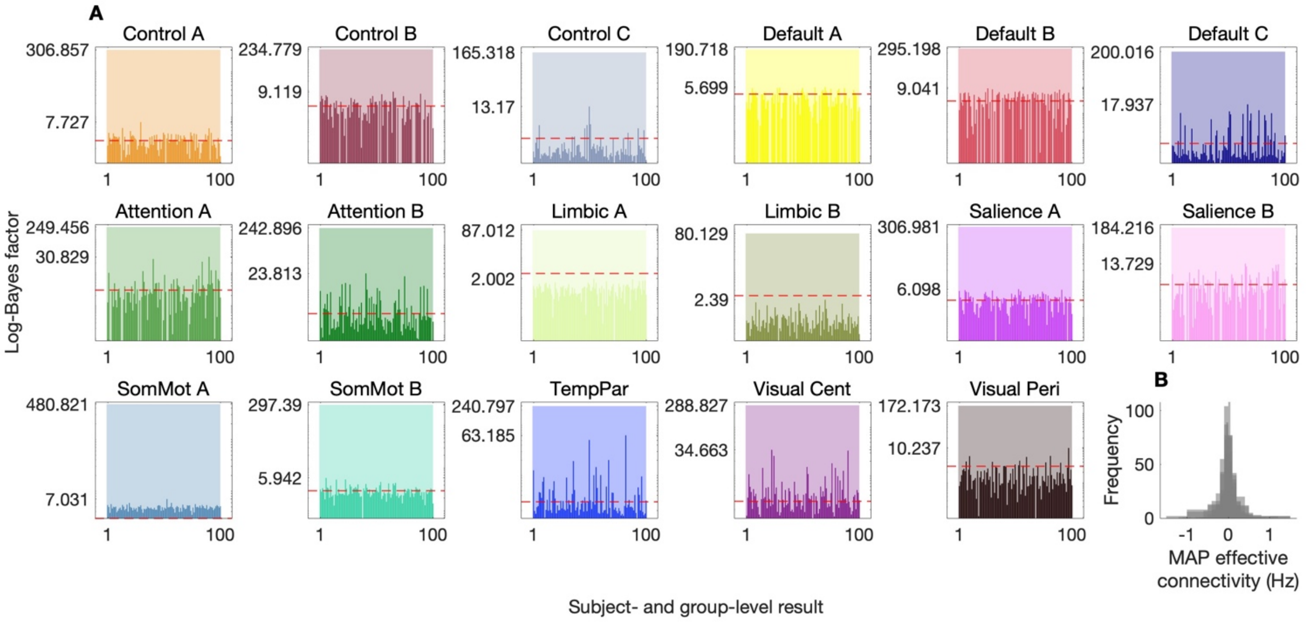

Fig. S2. Test-retest reliability of hierarchical empirical Bayes models. (**A**) Log-Bayes factors comparing hierarchical empirical Bayes models with structure-based priors (derived from out-of-session BMA prior-variance transformations) to models with non-informative priors, across 17 effective connectivity brain networks. The semi-transparent bars represent the log-Bayes factor for group-level component of the model, while the opaque bars represent the log-Bayes factor for the subject-level component. The log-Bayes factors indicate substantially greater evidence for models incorporating structure-based priors. Note the use of a log-scaled y-axis, with a dashed red line indicating a log-Bayes factor of 3. Y-axis tick marks indicate the maximum increase in evidence at the subject level, and the increase in evidence at the group level. Log-scaling necessitated the exclusion of several decreases in evidence at the subject level (see Figs. S2 for alternative visualizations). Networks analyzed include control (Control A–C), default mode (Default A–C), attention (Attention A, B), limbic (Limbic A, B), salience (Salience A, B), somatomotor (SomMot A, B), temporoparietal (TempPar), and visual networks (Visual Cent, central; Visual Peri, peripheral). (**B**) Histogram of maximum a posteriori (MAP) group-level effective connectivity estimates for all networks.

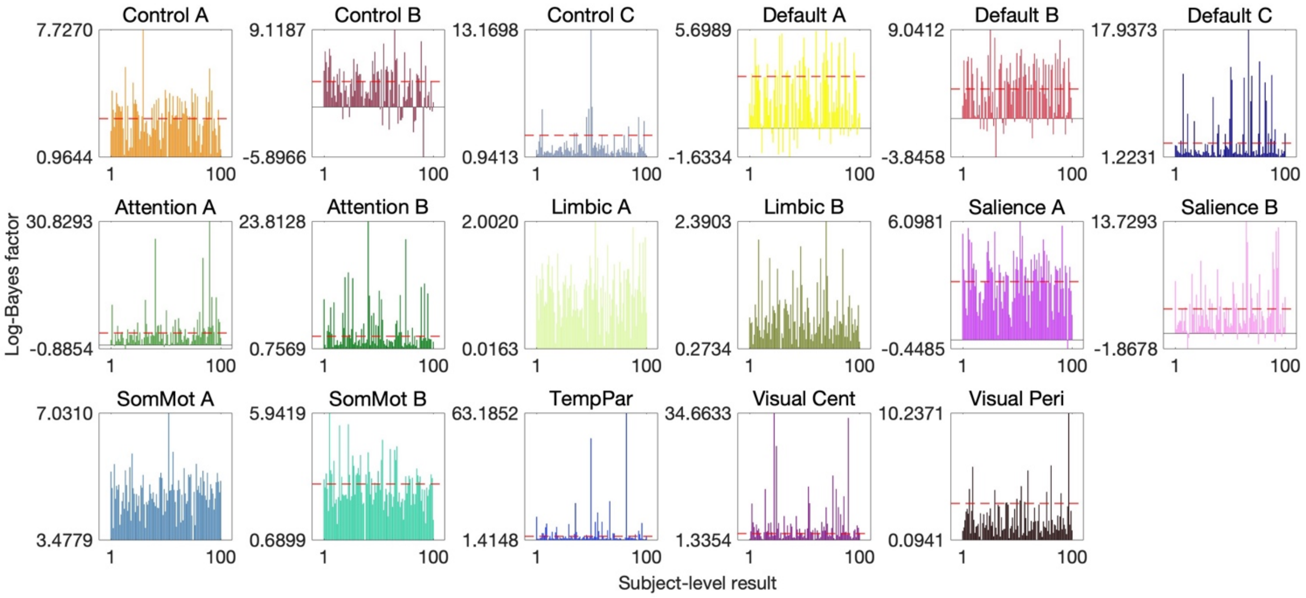

Fig. S3. Test-retest reliability of hierarchical empirical Bayes models (alternative visualization). Log-Bayes factors comparing hierarchical empirical Bayes models with structure-based priors (derived from out-of-session BMA prior-variance transformations) to models with non-informative priors, across 17 effective connectivity brain networks. The opaque bars represent the log-Bayes factor for the subject-level component of the model, indicating substantially greater evidence for models incorporating structure-based priors. The horizontal dashed red line indicates a log-Bayes factor of 3. Y-axis tick marks indicate the maximum increase in evidence and the minimum increase (or maximum decrease) in evidence. Networks analyzed include control (Control A–C), default mode (Default A–C), attention (Attention A, B), limbic (Limbic A, B), salience (Salience A, B), somatomotor (SomMot A, B), temporoparietal (TempPar), and visual networks (Visual Cent, central; Visual Peri, peripheral).

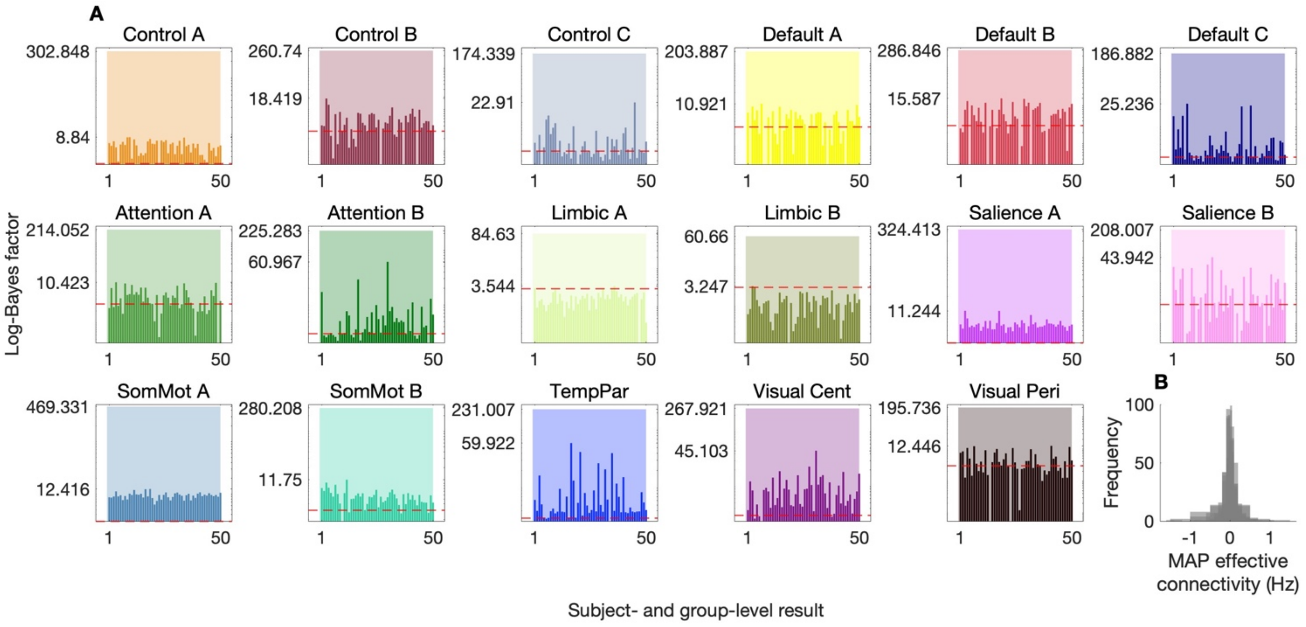

Fig. S4. Out-of-sample validation of hierarchical empirical Bayes models (session-2 results). (**A**) Log-Bayes factors comparing hierarchical empirical Bayes models with structure-based priors (derived from out-of-sample BMA prior-variance transformations) to models with non-informative priors, across 17 effective connectivity brain networks. The semi-transparent bars represent the log-Bayes factor for group-level component of the model, while the opaque bars represent the log-Bayes factor for the subject-level component. The log-Bayes factors indicate substantially greater evidence for models incorporating structure-based priors. Note the use of a log-scaled y-axis, with a dashed red line indicating a log-Bayes factor of 3. Y-axis tick marks indicate the maximum increase in evidence at the subject level, and the increase in evidence at the group level. Log-scaling necessitated the exclusion of several decreases in evidence at the subject level (see Figs. S4 for alternative visualizations). Networks analyzed include control (Control A–C), default mode (Default A–C), attention (Attention A, B), limbic (Limbic A, B), salience (Salience A, B), somatomotor (SomMot A, B), temporoparietal (TempPar), and visual networks (Visual Cent, central; Visual Peri, peripheral). (**B**) Histogram of maximum a posteriori (MAP) group-level effective connectivity estimates for all networks.

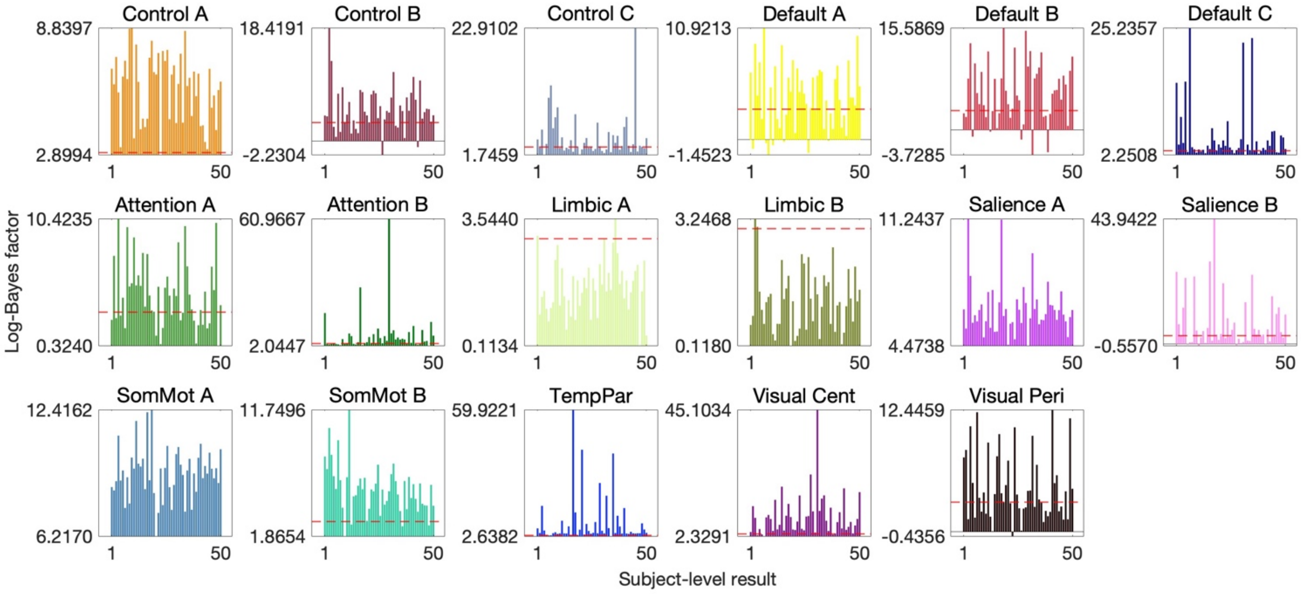

Fig. S5. Out-of-sample validation of hierarchical empirical Bayes models (session-2 results; alternative visualization). Log-Bayes factors comparing hierarchical empirical Bayes models with structure-based priors (derived from out-of-sample BMA prior-variance transformations) to models with non-informative priors, across 17 effective connectivity brain networks. The opaque bars represent the log-Bayes factor for the subject-level component of the model, indicating substantially greater evidence for models incorporating structure-based priors. The horizontal dashed red line indicates a log-Bayes factor of 3. Y-axis tick marks indicate the maximum increase in evidence and the minimum increase (or maximum decrease) in evidence. Networks analyzed include control (Control A–C), default mode (Default A–C), attention (Attention A, B), limbic (Limbic A, B), salience (Salience A, B), somatomotor (SomMot A, B), temporoparietal (TempPar), and visual networks (Visual Cent, central; Visual Peri, peripheral).

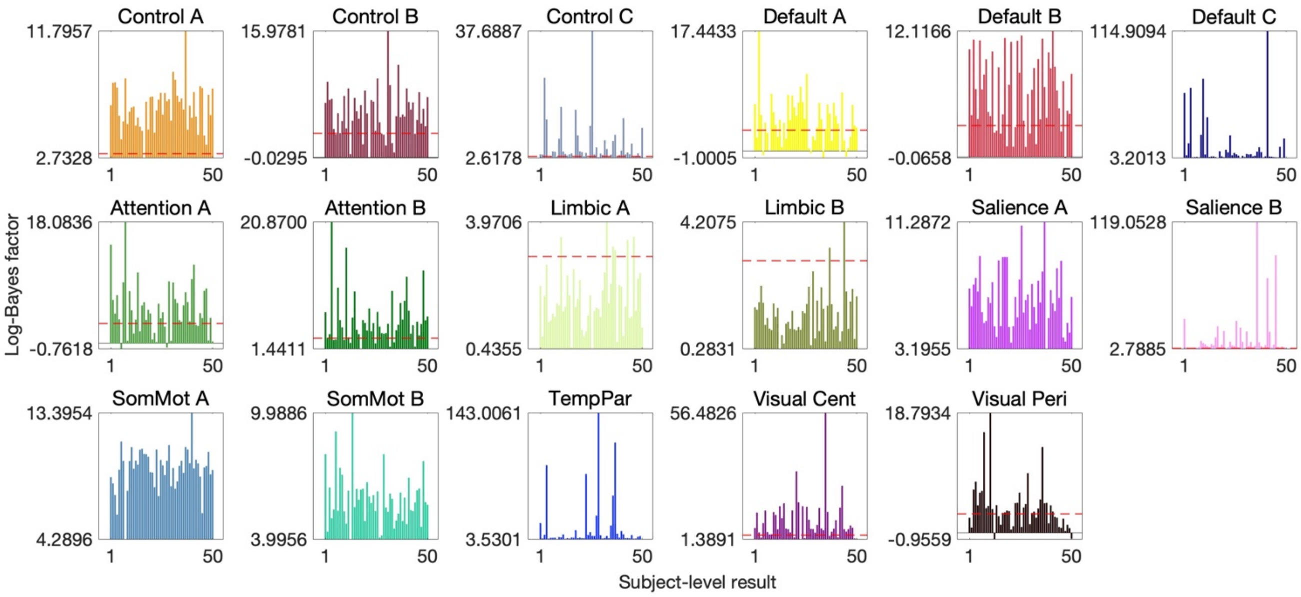

Fig. S6. Out-of-sample validation of hierarchical empirical Bayes models (session-1 results; alternative visualization). Log-Bayes factors comparing hierarchical empirical Bayes models with structure-based priors (derived from out-of-sample BMA prior-variance transformations) to models with non-informative priors, across 17 effective connectivity brain networks. The opaque bars represent the log-Bayes factor for the subject-level component of the model, indicating substantially greater evidence for models incorporating structure-based priors. The horizontal dashed red line indicates a log-Bayes factor of 3. Y-axis tick marks indicate the maximum increase in evidence and the minimum increase (or maximum decrease) in evidence. Networks analyzed include control (Control A–C), default mode (Default A–C), attention (Attention A, B), limbic (Limbic A, B), salience (Salience A, B), somatomotor (SomMot A, B), temporoparietal (TempPar), and visual networks (Visual Cent, central; Visual Peri, peripheral).

References

1. Friston, K., Mattout, J., Trujillo-Barreto, N., Ashburner, J. & Penny, W. Variational free energy and the Laplace approximation. *NeuroImage* **34**, 220–234 (2007).
2. Friston, K. J. *et al.* Bayesian model reduction and empirical Bayes for group (DCM) studies. *NeuroImage* **128**, 413–431 (2016).
3. Friston, K. J., Ashburner, J. T., Kiebel, S. J., Nichols, T. E. & Penny, W. D. *Statistical Parametric Mapping: The Analysis of Functional Brain Images*. (Elsevier Science, Burlington, 2011).
4. Stephan, K. E., Weiskopf, N., Drysdale, P. M., Robinson, P. A. & Friston, K. J. Comparing hemodynamic models with DCM. *NeuroImage* **38**, 387–401 (2007).
5. Stephan, K. E., Tittgemeyer, M., Knösche, T. R., Moran, R. J. & Friston, K. J. Tractography-based priors for dynamic causal models. *NeuroImage* **47**, 1628–1638 (2009).
6. Sokolov, A. A. *et al.* Linking structural and effective brain connectivity: structurally informed Parametric Empirical Bayes (si-PEB). *Brain Struct Funct* **224**, 205–217 (2019).
7. Sokolov, A. A. *et al.* Asymmetric high-order anatomical brain connectivity sculpts effective connectivity. *Network Neuroscience* **4**, 871–890 (2020).
8. Friston, K. J., Kahan, J., Biswal, B. & Razi, A. A DCM for resting state fMRI. *NeuroImage* **94**, 396–407 (2014).
9. Marsaglia, G. & Tsang, W. W. The Ziggurat Method for Generating Random Variables. *J. Stat. Soft.* **5**, (2000).
10. Razi, A., Kahan, J., Rees, G. & Friston, K. J. Construct validation of a DCM for resting state fMRI. *NeuroImage* **106**, 1–14 (2015).
11. Van Essen, D. C. *et al.* The WU-Minn Human Connectome Project: An overview. *NeuroImage* **80**, 62–79 (2013).
12. Smith, S. M. *et al.* Resting-state fMRI in the Human Connectome Project. *NeuroImage* **80**, 144–168 (2013).

13. Glasser, M. F. *et al.* The minimal preprocessing pipelines for the Human Connectome Project. *NeuroImage* **80**, 105–124 (2013).
14. Mori, S., Crain, B. J., Chacko, V. P. & Van Zijl, P. C. M. Three-dimensional tracking of axonal projections in the brain by magnetic resonance imaging. *Annals of Neurology* **45**, 265–269 (1999).
15. Tournier, J.-D. *et al.* MRtrix3: A fast, flexible and open software framework for medical image processing and visualisation. *NeuroImage* **202**, 116137 (2019).
16. Smith, R. E., Tournier, J.-D., Calamante, F. & Connelly, A. SIFT2: Enabling dense quantitative assessment of brain white matter connectivity using streamlines tractography. *NeuroImage* **119**, 338–351 (2015).
17. Smith, R. E., Tournier, J.-D., Calamante, F. & Connelly, A. Anatomically-constrained tractography: Improved diffusion MRI streamlines tractography through effective use of anatomical information. *NeuroImage* **62**, 1924–1938 (2012).
18. Schaefer, A. *et al.* Local-Global Parcellation of the Human Cerebral Cortex from Intrinsic Functional Connectivity MRI. *Cereb Cortex* **28**, 3095–3114 (2018).
19. Thomas Yeo, B. T. *et al.* The organization of the human cerebral cortex estimated by intrinsic functional connectivity. *Journal of Neurophysiology* **106**, 1125–1165 (2011).
20. Gajwani, M. *et al.* Can hubs of the human connectome be identified consistently with diffusion MRI? *Network Neuroscience* **7**, 1326–1350 (2023).
21. Betzel, R. F., Griffa, A., Hagmann, P. & Mišić, B. Distance-dependent consensus thresholds for generating group-representative structural brain networks. *Network Neuroscience* **3**, 475–496 (2019).
22. Margulies, D. S. *et al.* Situating the default-mode network along a principal gradient of macroscale cortical organization. *Proc. Natl. Acad. Sci. U.S.A.* **113**, 12574–12579 (2016).
23. Mesulam, M. From sensation to cognition. *Brain* **121**, 1013–1052 (1998).
