# Supplementary material for: Structurally informed resting-state effective connectivity recapitulates cortical hierarchy
Source: bioRxiv. 2024 Apr 4:2024.04.03.587831. Preprint. [Version 1] doi: 10.1101/2024.04.03.587831 (PMC11014588; doi:10.1101/2024.04.03.587831)
Supplement: Supplementary file 1 [file media-1.pdf]

Supplementary Material

Structurally informed resting-state effective connectivity recapitulates cortical hierarchy

Matthew D. Greaves[1,2*], **Leonardo Novelli**[1,2], **and Adeel Razi**[1,2,3,4]

[1]Turner Institute for Brain and Mental Health, Monash University, Clayton, 3800, Australia

[2]Monash Biomedical Imaging, Monash University, Clayton, 3800, Australia

[3]Wellcome Centre for Human Neuroimaging, University College London, London, WC1N 3AR, United Kingdom

[4]CIFAR Azrieli Global Scholars Program, CIFAR, Toronto, M5G 1M1, Canada

*matthew.greaves@monash.edu

Alternative visualizations

In the manuscript, log scaling applied to Fig. 3a and Fig 4a necessitated the exclusion of several decreases in log evidence at the subject level. Here, we provide two alternative visualizations—for the test-retest and session-1 out-of-sample validation results—utilizing the (raw) log evidence estimates.

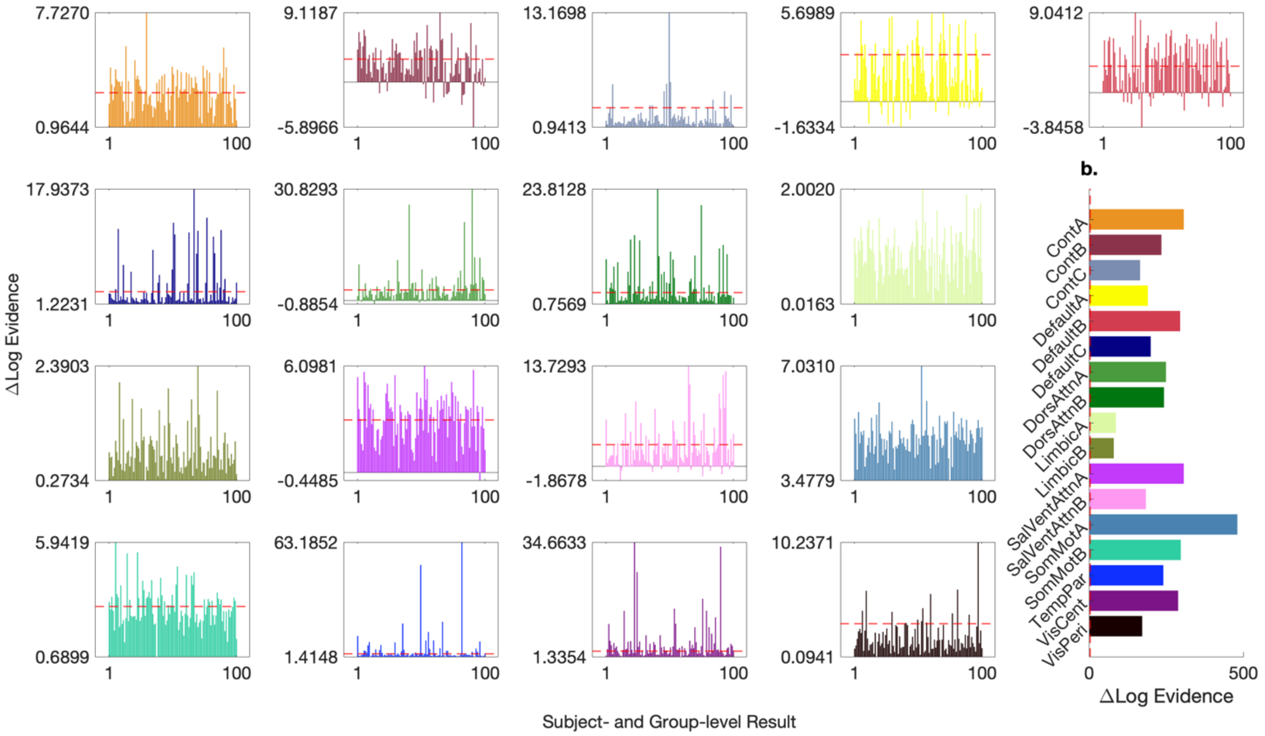

Supplementary Fig 1. Test-retest reliability of hierarchical empirical Bayes models for the 17 Schaefer brain networks (100 subjects). **a** Bar plots show relative log evidence for hierarchical empirical Bayes models evaluated—both steps 1 and 2—after applying the Bayesian model-average variance transformations identified out of session. Each bar represents the difference in log evidence—at the subject level—between hierarchical empirical Bayes models which utilize empirical constraints—structure-based priors—at the third level, and null (structurally uninformed) models which utilize generic priors at the third level. A dashed red line indicates a relative log evidence (or log Bayes factor) of 3, considered strong evidence against the null.[1] Tick marks on the y-axis indicate the minimum and maximum relative log evidence at the subject level. **b** Bar plot presents a side-by-side comparison of each relative log evidence for hierarchical empirical Bayes models (the group-level difference in log evidence). Again, a dashed red line indicates a relative log evidence of 3, and tick marks on the y-axis show Schafer network abbreviations (we refer the reader to the network parcellation subsection of the Methods section for network nomenclature). The color scheme for the bars follows a standardized palette and is applied to the plots in the first panel.

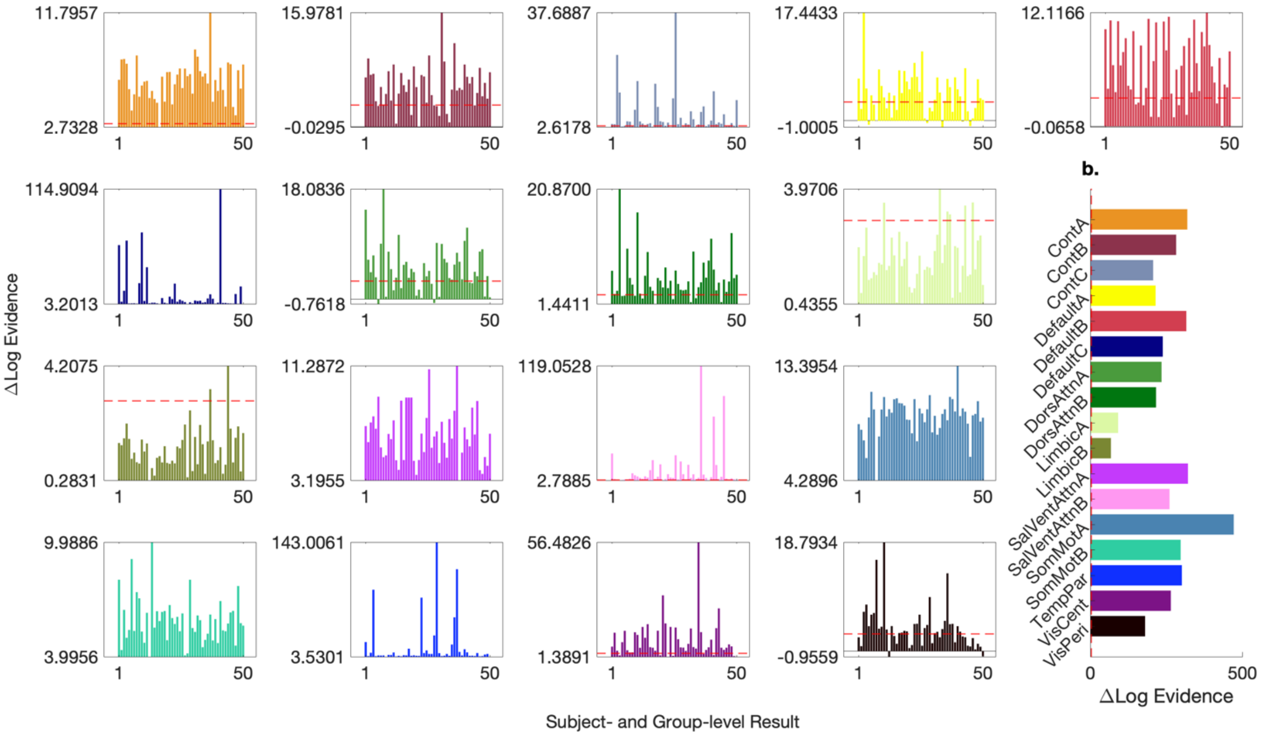

Supplementary Fig 2. Out-of-sample validation of hierarchical empirical Bayes models for the 17 Schaefer brain networks (50 subjects). **a** Bar plots show relative log evidence for hierarchical empirical Bayes models evaluated—both steps 1 and 2—after applying the Bayesian model-average variance transformations identified out of sample. Each bar represents the difference in log evidence—at the subject level—between hierarchical empirical Bayes models which utilize empirical constraints—structure-based priors—at the third level, and null (structurally uninformed) models which utilize generic priors at the third level. A dashed red line indicates a relative log evidence (or log Bayes factor) of 3, considered strong evidence against the null.[1] Tick marks on the y-axis indicate the minimum and maximum relative log evidence at the subject level. **b** Bar plot presents a side-by-side comparison of each relative log evidence for hierarchical empirical Bayes models (the group-level difference in log evidence). Again, a dashed red line indicates a relative log evidence of 3, and tick marks on the y-axis show Schafer network abbreviations (we refer the reader to the network parcellation subsection of the Methods section for network nomenclature). The color scheme for the bars follows a standardized palette and is applied to the plots in the first panel.

Between-subject out-of-sample validation

We assessed the degree to which the network-specific BMA variance transformations identified in the exploratory, face-validation dataset served as a network-specific link between structural connectivity and prior variances over group-level effective connectivity. We conducted two out-of-sample

validations (in both validation subjects' session 1 and session 2 data). In this section, we present the out-of-sample validation results for session 2, and refer the reader to the Results section for the (similar) session 1 results.

Supplementary Fig. 3a indicates that the network-specific variance transformations yielded structural connectivity-based priors that, again, substantially improved the log evidence of group-level effective connectivity models. Supplementary Fig. 3b shows that the pattern of increases in log evidence is consistent with that reported in the manuscript: increases are highest in one somatomotor (SomMotA) network, and lowest in one limbic (LimbicB) network. Again, for most subjects within most networks, the introduction of these out-of-sample network-specific BMA variance transformations translated to an increase in log evidence at the subject level. Supplementary Fig. 4 provides an alternative vizualisation of these results.

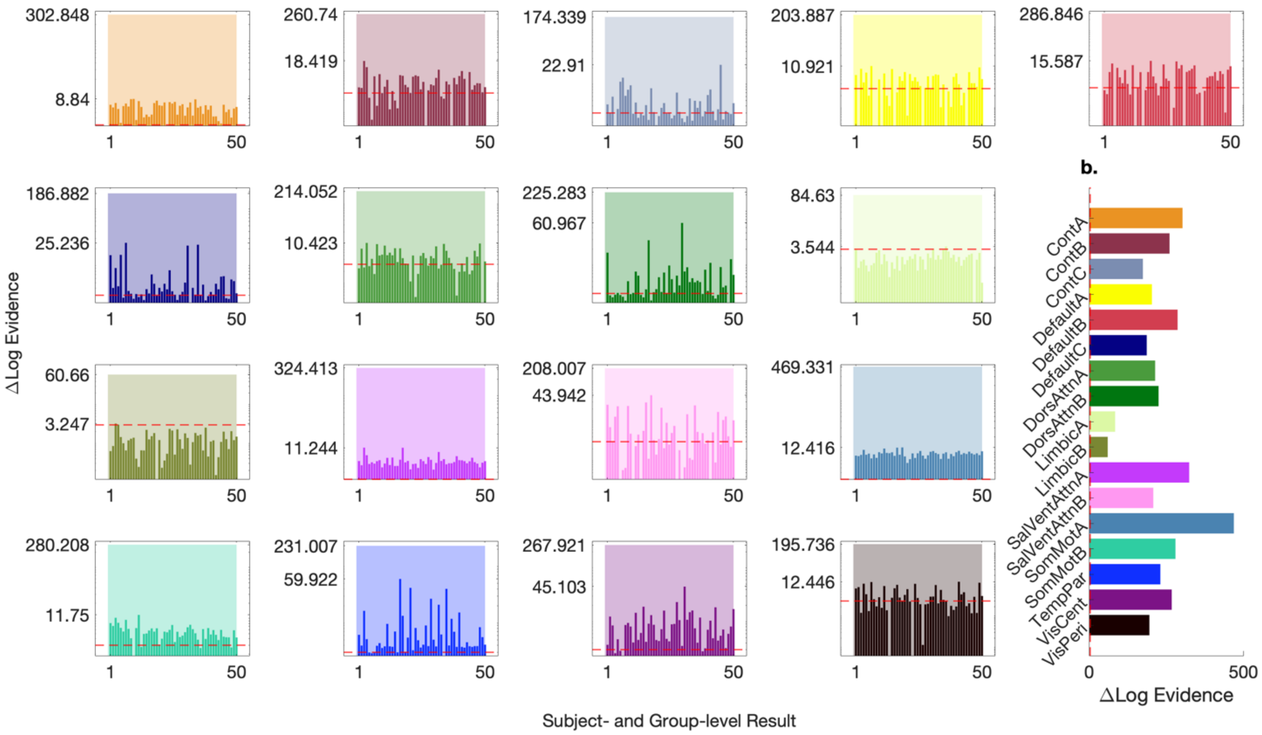

Supplementary Fig 3. Session-2 out-of-sample validation of hierarchical empirical Bayes models for the 17 Schaefer brain networks (50 subjects). **a** Bar plots show relative log evidence for hierarchical empirical Bayes models evaluated—both steps 1 and 2—after applying the Bayesian model-average variance transformations identified out of sample. Each bar represents the difference in log evidence—at the group (semitransparent bars) and subject (opaque bars) level—between hierarchical empirical Bayes models which utilize empirical constraints—structure-based priors—at the third level, and null (structurally uninformed) models which utilize generic priors at the third level. Note the use of a log-scaled y-axis, with a dashed red line indicating a relative log

evidence (or log Bayes factor) of 3, considered strong evidence against the null.[1] Tick marks on the y-axis indicate the maximum increase in log evidence at the subject level, and the increase in log evidence at the group level. Log-scaling necessitated the exclusion of several decreases in log evidence at the subject level (see Supplementary Fig. 2 for alternative visualization). **b** Bar plot presents a side-by-side comparison of each relative log evidence for hierarchical empirical Bayes models (the group-level difference in log evidence). Again, a dashed red line indicates a relative log evidence of 3, and tick marks on the y-axis show Schafer network abbreviations (we refer the reader to the network parcellation subsection of the Methods section for network nomenclature). The color scheme for the bars follows a standardized palette and is applied to the plots in the first panel.

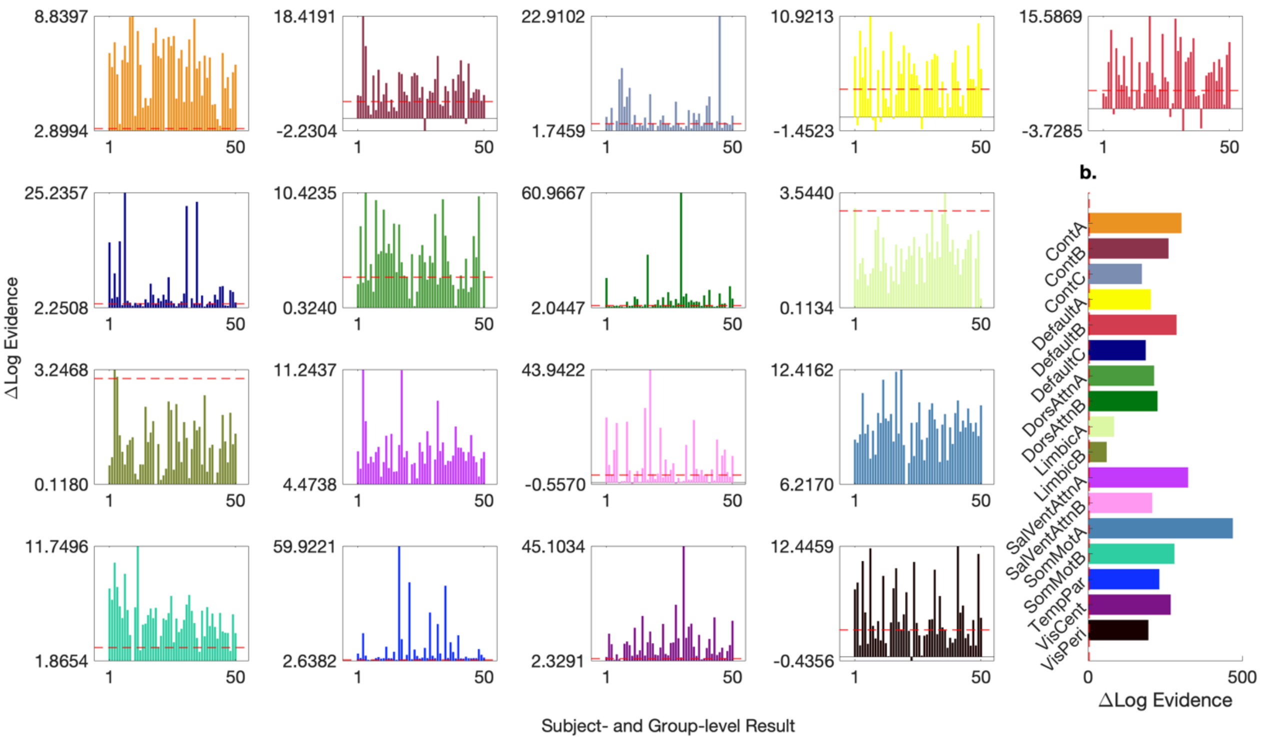

Supplementary Fig 4. Session-2 out-of-sample validation of hierarchical empirical Bayes models for the 17 Schaefer brain networks (50 subjects). **a** Bar plots show relative log evidence for hierarchical empirical Bayes models evaluated—both steps 1 and 2—after applying the Bayesian model-average variance transformations identified out of sample. Each bar represents the difference in log evidence—at the subject level—between hierarchical empirical Bayes models which utilize empirical constraints—structure-based priors—at the third level, and null (structurally uninformed) models which utilize generic priors at the third level. A dashed red line indicates a relative log evidence (or log Bayes factor) of 3, considered strong evidence against the null.[1] Tick marks on the y-axis indicate the minimum and maximum relative log evidence at the subject level. **b** Bar plot presents a side-by-side comparison of each relative log evidence for hierarchical empirical Bayes models (the group-level difference in log evidence). Again, a dashed red line indicates a relative log evidence of 3, and tick marks on the y-axis show Schafer network abbreviations (we refer the reader to the network parcellation

subsection of the Methods section for network nomenclature). The color scheme for the bars follows a standardized palette and is applied to the plots in the first panel.

References

1. Kass, R. E. & Raftery, A. E. Bayes Factors. *Journal of the American Statistical Association* **90**, 773–795 (1995).
